# Synthesis, Magnetic Properties, and Catalytic Properties of a Nickel(II)-Dependent Biomimetic of Metallohydrolases

**DOI:** 10.3389/fchem.2018.00441

**Published:** 2018-09-25

**Authors:** Adolfo Horn Jr., Daniel Englert, Asha E. Roberts, Peter Comba, Gerhard Schenk, Elizabeth H. Krenske, Lawrence R. Gahan

**Affiliations:** ^1^Laboratório de Ciências Químicas, Universidade Estadual do Norte Fluminense Darcy Ribeiro, Campos dos Goytacazes, Brazil; ^2^Anorganisch-Chemisches Institut and Interdisciplinary Center of Scientific Computing, Universität Heidelberg, Heidelberg, Germany; ^3^School of Chemistry and Molecular Biosciences, The University of Queensland, Brisbane, QLD, Australia

**Keywords:** nickel, phosphoesterase, magnetism, DFT, kinetics, mechanism

## Abstract

A dinickel(II) complex of the ligand 1,3-bis(bis(pyridin-2-ylmethyl)amino)propan-2-ol (HL1) has been prepared and characterized to generate a functional model for nickel(II) phosphoesterase enzymes. The complex, [Ni_2_(L1)(μ-OAc)(H_2_O)_2_](ClO_4_)_2_·H_2_O, was characterized by microanalysis, X-ray crystallography, UV-visible, and IR absorption spectroscopy and solid state magnetic susceptibility measurements. Susceptibility studies show that the complex is antiferromagnetically coupled with the best fit parameters *J* = −27.4 cm^−1^, *g* = 2.29, *D* = 28.4 cm^−1^, comparable to corresponding values measured for the analogous dicobalt(II) complex [Co_2_(L1)(μ-OAc)](ClO_4_)_2_·0.5 H_2_O (*J* = −14.9 cm^−1^ and *g* = 2.16). Catalytic measurements with the diNi(II) complex using the substrate bis(2,4-dinitrophenyl)phosphate (BDNPP) demonstrated activity toward hydrolysis of the phosphoester substrate with *K*_*m*_ ~10 mM, and *k*_*cat*_ ~0.025 s^−1^. The combination of structural and catalytic studies suggests that the likely mechanism involves a nucleophilic attack on the substrate by a terminal nucleophilic hydroxido moiety.

## Introduction

Our understanding of the bioinorganic significance of nickel can be traced to the discovery that the specific activity of the soluble jack bean urease, after partial EDTA-promoted inactivation, was a linear function of the nickel content, consistent with the presence of two nickel(II) ions per subunit of the pure enzyme (Dixon et al., 1980; Blakeley et al., 1982; Blakeley and Zerner, 1984). Previous to this discovery the importance of metal ions in general for the activity of urease was known, although the specific requirement for Ni(II) ions was not (Jacoby, 1933; Shaw, 1954; Shaw and Raval, 1961; Spears et al., 1977). Subsequently it was recognized that nickel is also required for the enzymatic activity of carbon monoxide dehydrogenase, (Ensign et al., 1989, 1990; Shin and Lindahl, 1992; Gencic and Grahame, 2003) and plays an important role in other bioinorganic systems, (Ashwini, 2006) including [NiFe]-hydrogenase (Przybyla et al., 1992; Sargent, 2016; Vaissier and Van, 2017) and a nickel dependent superoxide dismutase (Barondeau et al., 2004). In contrast to other bioinorganic systems Ni(II) complexes have received less attention. A limited number of studies have focussed on di-Ni(II) model complexes for urease (Meyer, 2009) and FeNi complexes for hydrogenases (Vaissier and Van, 2017), and Neves and colleagues have developed several Ni(II) models for phosphatases, in particular purple acid phosphatases (PAPs) (Greatti et al., 2008; Piovezan et al., 2012; Xavier and Neves, 2016). PAPs present an ideal system to study biomimetics, in parts because of the wealth of sequence (Flanagan et al., 2006), structural and functional data available (Schenk et al., 2013), but also because these enzymes occur in homo- and hetero-bimetallic form in nature, using a range of metal ions (Fe, Zn, Mn; Mitić et al., 2009, 2010). Furthermore, the Fe(III)Ni(II) form of PAP is catalytically active, one of the few known metallohydrolases that can accommodate Ni(II) and maintain functionality (Schenk et al., 2008). This flexibility of PAPs with respect to their use of metal ions may be a reflection of their dual function as a phosphatase and peroxidase; indeed, in its di-Fe(III) form PAP is easily and reversibly reduced to the heterovalent Fe(III)Fe(II) form (redox potential ~340 mV), a process that allows the enzyme to act as a Fenton catalyst (Sibille et al., 1987; Bernhardt et al., 2004).

**Figure 1:**
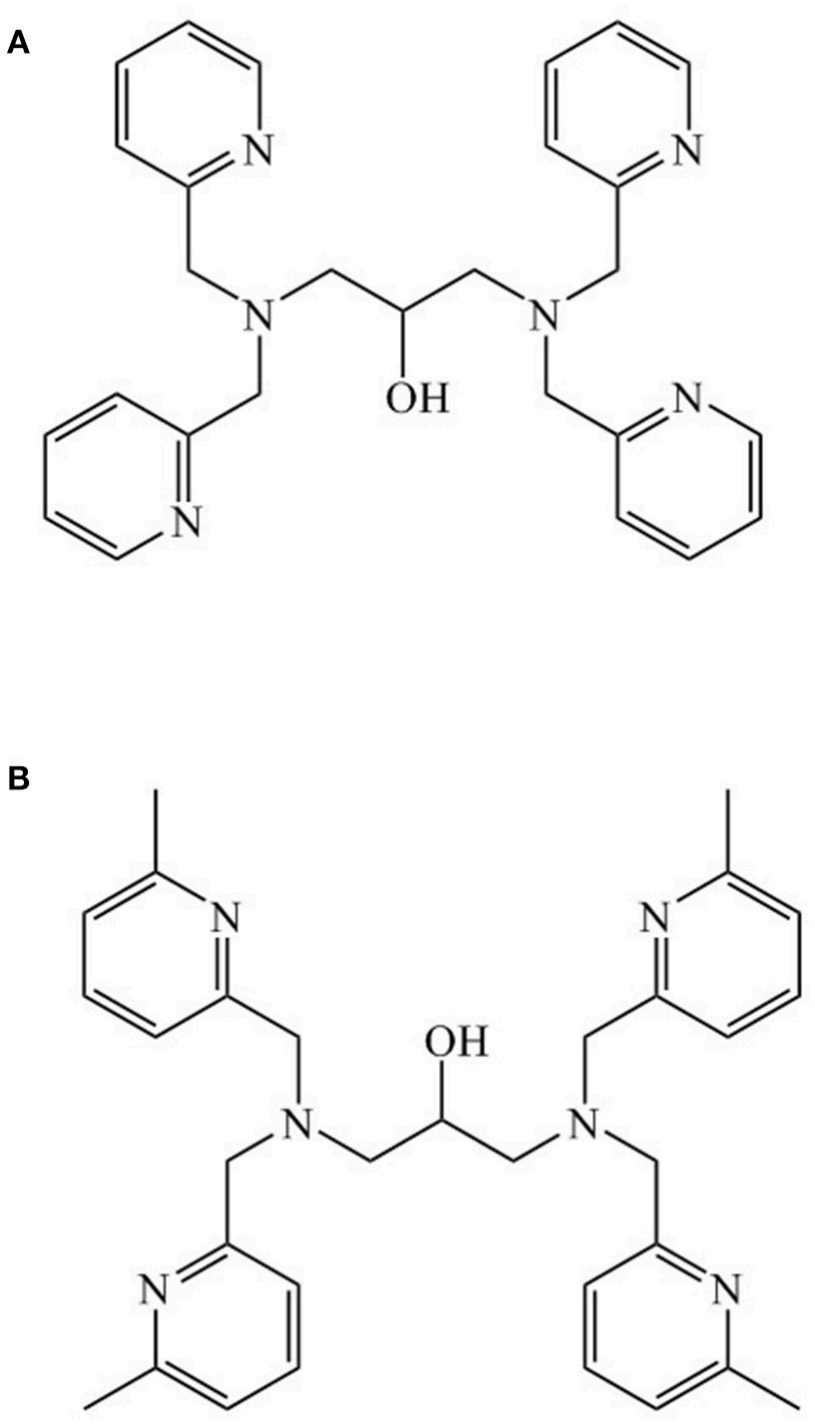
**(A)** 1,3-bis(bis(pyridin-2-ylmethyl)amino)propan-2-ol (HL1); **(B)** N,N,N',N'-tetrakis[(6-methyl-2-pyridyl)methyl]-1,3-diaminopropan-2-ol (Me4tpdpH).

The metal ion composition of PAP may also influence its reaction mechanism; in particular, the identity of the hydrolysis-initiating nucleophile may be affected by the identity of the metal ions (Mitić et al., 2010; Selleck et al., 2017). Here, we selected ligand 1,3-bis(bis(pyridin-2-ylmethyl)amino)propan-2-ol (Figure 1) to probe the possibility that a di-Ni(II) biomimetic may promote phosphatase activity. This ligand, previously used to generate a di-Mn(II) system (Suzuki et al., 1989; Sato et al., 1992), offers an opportunity to investigate the hydrolytic mechanism as it offers a limited number of possible pathways for the nucleophile.

## Experimental section

### General methods

Chemicals were purchased from Sigma-Aldrich, Merck, ABCR, Acros or Alfa Aeser and used without further purification. Reactions requiring the exclusion of moisture and/or oxygen were carried out under nitrogen atmosphere using standard Schlenk techniques. TLC was performed on TLC Silica gel 60 F_254_ TLC plates purchased from Merck and visualization of the spots was carried out by fluorescence quenching with 254 nm UV light. Purification of raw products by column chromatography were performed using silica gel (grade 9385, 60 Å, 230–400 mesh size) purchased from Sigma-Aldrich. NMR spectra were recorded with a Bruker Avance III 300 system at 300 K. Chemical shifts (δ) are given in ppm and coupling constants (*J)* in Hz. ^1^H and ^13^C spectra were referenced to the protio impurity or the ^13^C signal of the deuterated solvent. Abbreviations used for observed multiplicities are d for doublet, dd for doublet of doublets, td for triplet of doublets and m for multiplet. IR spectra were measured with a Perkin Elmer Frontier FT-IR spectrometer, transmittance data are given in wave number $\tilde{\nu }$ (cm^−1^). Abbreviations used for observed intensities are w for weak, m for medium and s for strong. UV-Vis absorption spectra were recorded with an Agilent Technologies Cary 60 UV-Vis spectrophotometer. Elemental analyses were performed by the elemental microanalysis service at the School of Chemistry & Molecular Biosciences of the University of Queensland. The synthesis of the cobalt(II) complex, [Co_2_(L1)(μ-OAc)](ClO_4_)_2_·0.5 H_2_O, is described in the Supplementary Material.

### Hydrolysis studies

Kinetic studies were conducted using a Varian Cary50 Bio UV/Visible spectrophotometer with a Peltier temperature controller (25°C) and 10-mm quartz cuvettes, and employing bis-(2,4-dinitrophenol)phosphate (BDNPP) as substrate. Assays were measured in a solvent system composed of 50:50 acetonitrile:buffer. An aqueous multicomponent buffer was employed made up of 50 mM 2-(N-morpholino)ethanesulfonic acid (MES), 4-(2-hydroxyethyl)-1-piperazineethanesulfonic acid (HEPES), 2-(N-cyclohexylamino)ethane sulfonic acid (CHES) and *N*-cyclohexyl-3-aminopropanesulfonic acid (CAPS), with ionic strength controlled with LiClO_4_ (250 mM). The pH values reported for the buffers are those of the aqueous component (Kaminskaia et al., 2000). The initial-rate method was employed and assays were measured such that the initial linear portion of the data was used for analysis. Product formation was determined by monitoring the formation of 2,4-dinitrophenolate; the extinction coefficient of this product at 400 nm varies from 7,180 at pH 4.5, 10,080 at pH 5.0; 11,400 at pH 5.5 to 12,000 at 6.0 and 12,100 at pH 6.5–11 (Smith et al., 2007). Assays to evaluate the pH dependence of the reaction contained 40 μM complex and 5 mM BDNPP; to evaluate the effect of [substrate], 0.5 mM complex was mixed with 1–11.5 mM BDNPP. Catalytic rates could be measured reliably up to pH 11.0 and were fit to the simplest possible model to describe the pH dependence of the observed catalytic rates (Kantacha et al., 2011). The model invokes one relevant protonation equilibrium and is described by an equation of the form

$$y(x)=\;\frac{a}{1+\;\frac{\text{x}}{\text{b}}}$$

Here, *a* and b represent fitting parameters (i.e., *V*_max_ and *K*_a_, respectively), while x is the variable ([H^+^] in this case) for a function of y (representing the measurable catalytic rate *v*). At very high [H^+^] (low pH; x/b>>0) the denominator is >> V_max_ and hence the rate is approximating 0. At very low [H^+^] (high pH; x/b~0) the denominator approximates 1 and hence the rate approaches V_max_. The fitting parameter *b*, representing the relevant acid dissociation constant *K*_a_, is thus the [H^+^] where the rate *v* reaches half of its maximum value V_max_.

The catalytic rates were measured as a function of substrate concentration. Experimental limitations (imposed by the solubility of the substrate) prevented accurate measurements above 6 mM. The data displayed hyperbolic behavior but saturation was not achieved. Consequently, the data were analyzed with a combination of non-linear regression and double-reciprocal linear fits, using the Michaelis-Menten equation

$$v_{0}=\;\frac{V_{max}\left[S\right]}{K_{m}+\left[S\right]},$$

where *V*_0_ is the initial rate, *V*_*max*_ is the maximum rate, *K*_*M*_ is the Michaelis constant, and [S] is the substrate concentration.

### Susceptibility measurements

The magnetic data were collected using an MPMS-XL 5T (Quantum Design) SQUID magnetometer. Fixed powder samples were prepared by pressing the powder into PTFE tape to prevent field-induced reorientation. Data were corrected for contributions of the sample holders and, using Pascal's constants (Bain and Berry, 2008), for the diamagnetic contributions of the samples. Effective magnetic moments were calculated using the relationship μ_eff_ = 2.828(χ_M_T)${}^{\frac{1}{2}}$.

### Crystallographic measurements

Crystallographic data for the complex were collected at 190(2) K using an Oxford Diffraction Gemini Ultra dual source (Mo and Cu) CCD diffractometer with Mo (λ_Kα_ = 0.71073 Å) radiation. The structure was solved by direct methods (SIR-92) and refined by full matrix least squares methods (SHELXL 97) based on F^2^ (Sheldrick, 1997), accessed through the WINGX 1.70.01 crystallographic collective package (Farrugia, 1999). Hydrogen atoms were fixed geometrically and not refined. X-ray data of the published structure was deposited with the Cambridge Crystallographic Data Centre, CCDC 1844565.

### Computational details

Geometry optimizations of the cations of [Ni_2_(L1)(μ-OAc)(H_2_O)_2_](ClO_4_)_2_·H_2_O and [Co_2_(L1)(μ-OAc)](ClO_4_)_2_·0.5H_2_O were undertaken with the Gaussian 09 set of programs (Frisch et al., 2013) starting from the X-ray structural data. The B3LYP functional (Becke, 1993), Noodleman's broken symmetry, (Noodleman et al., 1988) the TZV basis set (Schaefer et al., 1992). Orca 2.6.04 (Neese, 2012) was used for the magnetic coupling constant calculation essentially as described previously (Comba et al., 2009).

### Synthesis

#### 1,3-Bis(Bis(pyridin-2-ylmethyl)amino)Propan-2-ol (HL1)

2-(Chloromethyl)pyridine hydrochloride (4.00 eq, 6.01 g, 36.62 mmol) was dissolved in 3 mL distilled water and 15 mL of an aqueous 5 M NaOH solution was added while stirring. 1,3-diaminopropan-2-ol (1.00 eq, 825 mg, 9.15 mmol), 15 mL of 5 M NaOH solution and tetraoctylammonium bromide (0.02 eq, 100 mg, 183 μmol) were then added. The resulting red mixture was stirred at room temperature overnight. The reaction mixture was transferred into a separatory funnel with 40 mL chloroform, 40 mL of brine, the aqueous phase was extracted twice with 10 mL chloroform, and the combined organic layers washed with 50 mL water. The separated organic layer was dried over magnesium sulfate, filtered and concentrated under reduced pressure. The crude product was purified using a MeOH-equilibrated silica column (Merck) according to the manufacturer's instructions. After removal of the solvent under reduced pressure the product was obtained as orange oil (88%, 3.66 g, 8.05 mmol). ^1^H NMR (300 MHz, CDCl_3_): δ 8.49–8.45 (4H, m), 7.54 (4H, td, *J* = 11.5 Hz, *J* = 1.8 Hz), 7.37–7.31 (4H, m), 7.12–7.05 (4H, m), 4.03–3.92 (1H, m), 3.89 (4H, d, ^2^*J* = 14.7 Hz), 3.84 (4H, d, ^2^*J* = 14.7 Hz), 2.69 (2H, dd, ^2^*J* = 13.3 Hz, ^3^*J* = 4.1 Hz), 2.59 (2H, dd, ^2^*J* = 13.3 Hz, ^3^*J* = 7.8 Hz) ppm. ^13^C NMR (75 MHz, CDCl_3_): δ 159.3, 149.0, 136.5, 123.2, 122.1, 67.2, 60.8, 59.1 ppm.

### [Ni_2_(L1)(μ-OAc)(H_2_O)_2_](CLO_4_)_2_·H_2_O

Nickel(II) acetate tetrahydrate (2.00 eq, 109.5 mg, 440 μmol) was dissolved in 6 mL MeOH. Lithium perchlorate trihydrate (4.00 eq, 153.4 mg, 880 μmol) and 4 mL of a methanol solution of 1,3-bis(bis(pyridin-2-ylmethyl)amino)propan-2-ol (HL1) (1.00 eq, 100.0 mg, 220 mmol) were added and the reaction mixture stirred under reflux for 1 h. Upon cooling to room temperature, the solvent was removed under reduced pressure and the resulting product was recrystallized in a water/acetone mixture resulting in blue crystals (46%, 89.6 mg). IR: $\tilde{\nu }$ = 3489 (w), 3221 (w), 2801 (w), 1651 (m), 1606 (s), 1548 (s), 1482 (m), 1445 (s), 1427 (s), 1287 (m), 1161 (m), 1070 (s), 1056 (s), 1037 (s), 1023 (s), 990 (m), 928 (m), 886 (m), 760 (s), 620 (s) cm^−1^. UV/vis λ_max_ (ε), 950 nm (57 M^−1^cm^−1^), 590 nm (43 M^−1^cm^−1^). Calc. for C_29_H_38_Cl_2_N_6_ Ni_2_O_14_: C, 39.45; H, 4.34; N, 9.52 %. Found: C, 39.49; H, 4.13; N, 9.37%.

## Results and discussion

### Syntheses

The ligand 1,3-bis(bis(pyridin-2-ylmethyl)amino)propan-2-ol **(**HL1**)** was prepared by a modification of previously described procedures (Suzuki et al., 1989; Sato et al., 1992). The nomenclature HL1 indicates that the ligand is protonated at the hydroxido moiety and on formation of the complex the ligand is deprotonated and coordinates as the monoanion L1^−^. We have reported previously a diZn(II) complex with L1^−^ as a functional model for zinc(II) phosphoesterase enzymes (Mendes et al., 2016).

In this work the dinuclear nickel complex [Ni_2_(L1)(μ-OAc)(H_2_O)_2_](ClO_4_)_2_.H_2_O was synthesized from a reaction of HL1 with two equivalents of nickel(II) acetate in methanol solution in the presence of LiClO_4_. The nickel complex was obtained as bright blue crystals after recrystallization in a water/acetone mixture.

A complex with the same ligand and formulated as [Ni_2_(L1)(μ-OAc)_2_](PF_6_).MeOH has been reported previously (Moffat et al., 2014). In that case the synthesis involved the reaction of the ligand HL1, nickel(II) acetate in methanol, in the presence of triethylamine and NaPF_6_ under reflux. On standing at −18°C the pink crystals which initially formed were removed, the mother liquor collected and concentrated under vacuum and upon slow diffusion of diethylether on standing the deep blue crystals of [Ni_2_(L1)(μ-OAc)_2_](PF_6_). MeOH were collected and structurally characterized (Moffat et al., 2014). In addition, the Ni(II) complex of a similar ligand *N,N,N*',*N*'-tetrakis((6-methyl-2-pyridyl)methyl)-1,3-diaminopropan-2-ol (Me_4_tpdpH), prepared by reaction of nickel(II) acetate, NaClO_4_ with Me_4_tpdpH in methanol at room temperature, and crystallized from a methanol/diethyl ether solution as light green crystals, has also been reported. The complex was formulated as [Ni_2_(Me_4_tpdp)(μ-OAc)(ClO_4_)(CH_3_OH)](ClO_4_) (Yamaguchi et al., 1997, 2001). The synthesis and characterization of the cobalt complex [Co_2_(L1)(μ-OAc)](ClO_4_)_2_ has been reported previously (Siluvai and Murthy, 2009).

### Spectroscopy

The infrared spectrum of [Ni_2_(L1)(μ-OAc)(H_2_O)_2_](ClO_4_)_2_ displayed bands attributed to the asymmetric and the symmetric acetate stretch (*v*_*as*_ = 1,548 cm^−1^, *v*_*s*_ = 1,445 cm^−1^), indicating the presence of a bridging acetate anion (Deacon and Phillips, 1980). Furthermore, characteristic bands at 1,606 cm^−1^ and 1,576 cm^−1^ were assigned to *v*_C = N_ and *v*_C = C_ of the pyridyl groups of the ligand, and those 1,070 cm^−1^ to the perchlorate counter ion.

The electronic spectrum of [Ni_2_(L1)(μ-OAc)(H_2_O)_2_](ClO_4_)_2_ was measured in acetonitrile. For octahedrally coordinated Ni(II) ions there are three spin allowed d-d transitions from the ^3^A_2g_ ground state to the higher excited triplet states ^3^T_2g_, ^3^T_1g_, and ^3^T_1g_ (P). Spectral bands with maxima at 950 and 590 nm were assigned to the ^3^A_2g_→^3^T_2g_ and ^3^A_2g_→^3^T_1g_ transitions, respectively. The ^3^A_2g_→^3^T_1g_ (P) transition is probably hidden under the intense band of the pyridyl groups with a maximum at 255 nm; a small shoulder around 400 nm could arise from this spin-allowed transition. A shoulder around 800 nm is assigned to the spin-forbidden d-d ^3^A_2g_→^1^E_1g_ transition. The Racah parameter B and the ligand field splitting energy Dq of the d^8^ Ni(II) system were determined using the bands of the ^3^A_2g_→^3^T_2g_ and ^3^A_2g_→^3^T_1g_ transitions and determined to be 877 and 1,053 cm^−1^, respectively.

### X-Ray crystal structure

**Figure 2:**
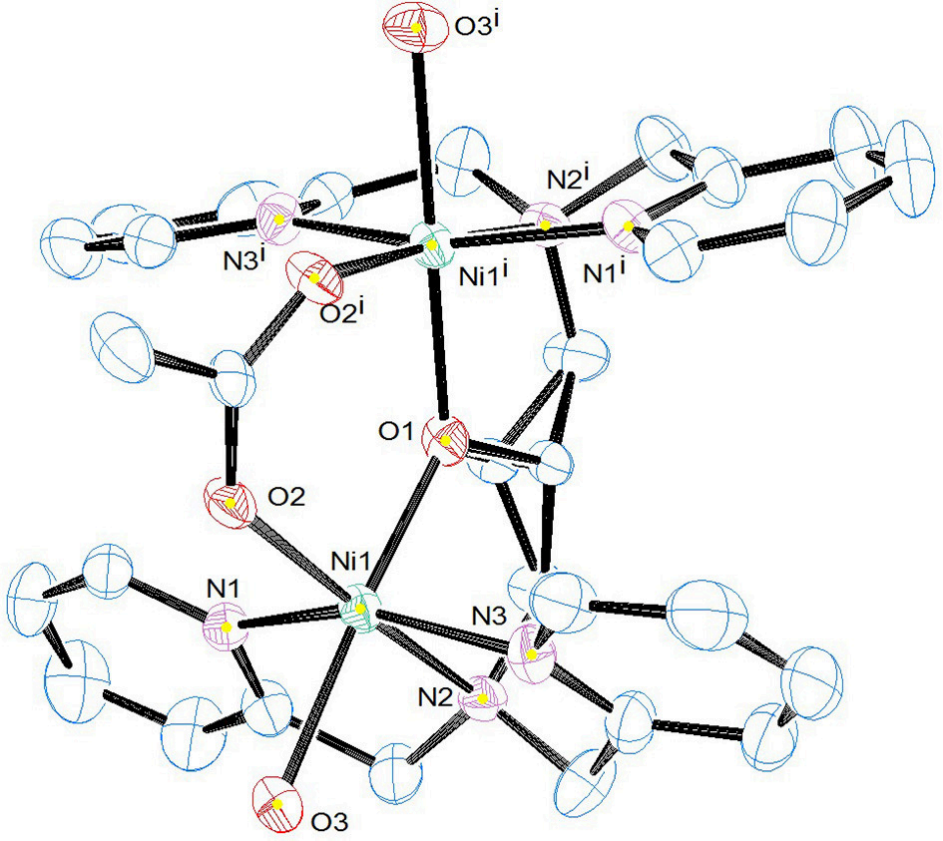
ORTEP plot of the cation [Ni_2_(L1)( μ-OAc)(H_2_O)_2_]^2+^. Counter ions and hydrogen atoms have been omitted for clarity (25% ellipsoid probability in ORTEP plot).

Selected crystallographic data for [Ni_2_(L1)(μ-OAc)(H_2_O)_2_](ClO_4_)_2_·H_2_O are shown in Table 1 and selected bond lengths and angles are displayed in Table 2. An ORTEP plot is shown in Figure 2. The structure is composed of the ligand mono-anion, two metal(II) ions and a bridging acetate with the Ni(II) structure containing two coordinated aqua ligands completing the hexacoordinate coordination sphere. The charge is balanced by perchlorate ions. In the diNi(II) complex there is disorder around the carbon atom of the bridging hydroxide. The coordination symmetry around each Ni(II) site for [Ni_2_(L1)(μ-OAc)(H_2_O)_2_](ClO_4_)_2_·H_2_O is octahedral and the donor set of both nickel atoms is N_3_O_3_, which is composed of a tertiary amino-N atom (Ni(1)-N(2): 2.110(4) Å), two pyridyl-N atoms (Ni(1)-N(1): 2.078(3) Å; Ni(1)-N(3): 2.080(4) Å), a bridging alkoxo-O atom (Ni(1)-O(1): 1.990(2) Å), an μ-acetato O atom (Ni(1)-O(2): 2.058(3) Å) and an O atom of a coordinating water molecule (Ni(1)-O(3): 2.132(3) Å). The bridging angle Ni-O(1)-Ni is 131.6° and the two nickel atoms are separated by a distance of 3.63 Å. The C(8) atom is disordered between two different positions.

**Table 1:** Crystallographic data for [Ni_2_(L1)(μ-OAc)(H_2_O)_2_](ClO_4_)_2_·H_2_O.

**Complex**	**[Ni_2_(L1)(μ-OAc)(H_2_O)_2_](ClO_4_)_2_·H_2_O**
Empirical formula	C_29_H_36_Cl_2_N_6_Ni_2_O_13_
Formula weight	864.96
Wavelength (Å)	0.71073 (Mo K_α_)
Crystal system	Monoclinic
Space group	C 2/c
*a* (Å)	12.7975(10)
*b* (Å)	21.0734(13)
*c* (Å)	13.8612(8)
α (°)	90
β (°)	105.169(7)
γ (°)	90
Vol (Å^3^)	3,607.9(4)
Z	4
μ (mm^−1^)	1.263
*F*(000)	1,784
ρ (Mg/m^3^)	1.592
Reflns col.	8510
Ind. Reflns (*R_*int*_*)	3,178(0.0533)
θ range (°)	3.48 to 25.00
GOOF on F^2^	1.042
final R indices [I>2σ(I)]	R1 = 0.0523, wR2 = 0.1171
R indices (all data)	R1 = 0.0733, wR2 = 0.1312

**Table 2:** Selected bond lengths (Å) and angles (°) for [Ni_2_(L1)(μ-OAc)(H_2_O)_2_](ClO_4_)_2_·H_2_O.

N(1)-Ni(1) 2.078(3)	N(2)-Ni(1) 2.110(4)	N(3)-Ni(1) 2.080(4)
O(1)-Ni(1) 1.990(2)	O(2)-Ni(1) 2.058(3)	O(3)-Ni(1) 2.132(3)
Ni(1)-O(1)-Ni(1) 131.6(2)	O(1)-Ni(1)-O2 93.57(13)	O(1)-Ni(1)-N(1) 96.25(10)
O(2)-Ni(1)-N(1) 97.99(13)	O(1)-Ni(1)-N(3) 92.40(10)	O(2)-Ni(1)-N(3) 99.49(13)
N(1)-Ni(1)-N(3) 159.95(14)	O(1)-Ni(1)-N(2) 83.55(14)	O(2)-Ni(1)-N(2) 176.71(13)
N(1)-Ni(1)-N(2) 80.79(14)	N(3)-Ni(1)-N(2) 82.26(14)	O(1)-Ni(1)-O(3) 176.18(12)
O(2)-Ni(1)-O(3) 89.54(12)	N(1)-Ni(1)-O(3) 85.50(13)	N(3)-Ni(1)-O(3) 84.89(12)
N(2)-Ni(1)-O(3) 93.40(13)		


**Figure 3:**
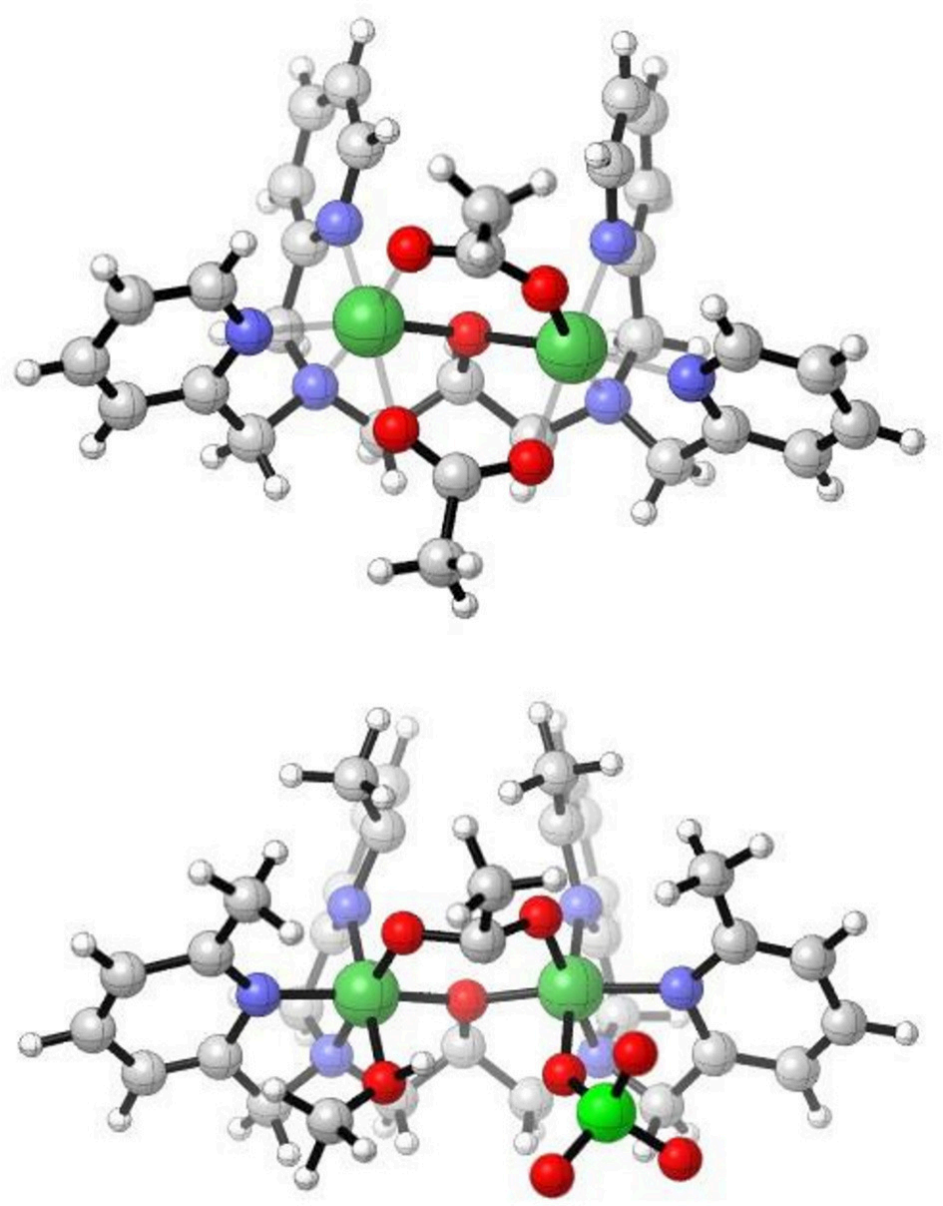
Cations of **(Top)** [Ni_2_L1(μ-OAc)_2_]^+^ (Moffat et al., 2014), and **(Bottom)** [Ni_2_(Me_4_tpdp)(μ-OAc)(ClO_4_)(CH_3_OH)]^+^ from the X-ray structures (Yamaguchi et al., 1997, 2001).

For the previously reported complex [Ni_2_(L1)(μ-OAc)_2_](PF_6_).MeOH, the two Ni(II) octahedral sites are composed of two μ-acetate ligands, the three N donors of the L1 ligand, and the μ-O of the ligand (Figure 3, top; Moffat et al., 2014). The differences in the two diNi(II) structures of L1^−^ involve the absence of the second bridging acetate ligand and the presence of the two terminal water molecules in [Ni_2_(L1)(μ-OAc)(H_2_O)_2_](ClO_4_)_2_·H_2_O, as well as the presence of the ClO_4_^−^ anions. Furthermore, the coordinating groups present in the ligand arms show a meridional coordination mode, which induces the coordination of the water molecules in positions *anti* to each other.

The X-ray crystal structure of [Ni_2_(Me_4_tpdp)(μ-OAc)(ClO_4_)(CH_3_OH)](ClO_4_) has been reported (Figure 3, bottom; Yamaguchi et al., 1997, 2001). The structure shows that both Ni(II) sites are six coordinate, the two metal ions separated by 3.62 Å and bridged by the ligand μ-O alkoxide and μ-acetato ligands. The sixth coordination site of one Ni(II) is made up of the O from a perchlorato ligand, whilst for the other metal ion a CH_3_OH ligand completes the sixth coordination (Yamaguchi et al., 1997, 2001). Whereas for the complex [Ni_2_(L1)(μ-OAc)(H_2_O)_2_](ClO_4_)_2_·H_2_O the coordinated aqua ligands are *trans* to the μ-alkoxo-O atom, effectively bisecting the two pyridyl rings of the ligand, this arrangement is not observed in the structure of the analogous [Ni_2_(Me_4_tpdp)(μ-OAc)(ClO_4_)(CH_3_OH)](ClO_4_) complex. Here, the methanol and perchlorato ligands are located *cis* to the μ-alkoxo-O atom (Yamaguchi et al., 1997), and presumably replaced by aqua ligands in solution, an important consideration in subsequent hydrolytic studies (Yamaguchi et al., 2001).

The X-ray crystal structure of [Co_2_(L1)(μ-OAc)](ClO_4_)_2_ has been reported (Siluvai and Murthy, 2009). The structure shows a pseudo C_2_-axis of symmetry with the μ-acetate ligand bridging the two Co(II) ions in a symmetric μ-1,3 mode. The two Co(II) sites display slightly distorted trigonal bipyramidal geometry with τ_av_, the index of trigonality, equal to 0.93; a τ value of unity would perfect trigonal bipyramidal geometry (Addison et al., 1984; Siluvai and Murthy, 2009).

### Magnetic susceptibility

**Figure 4:**
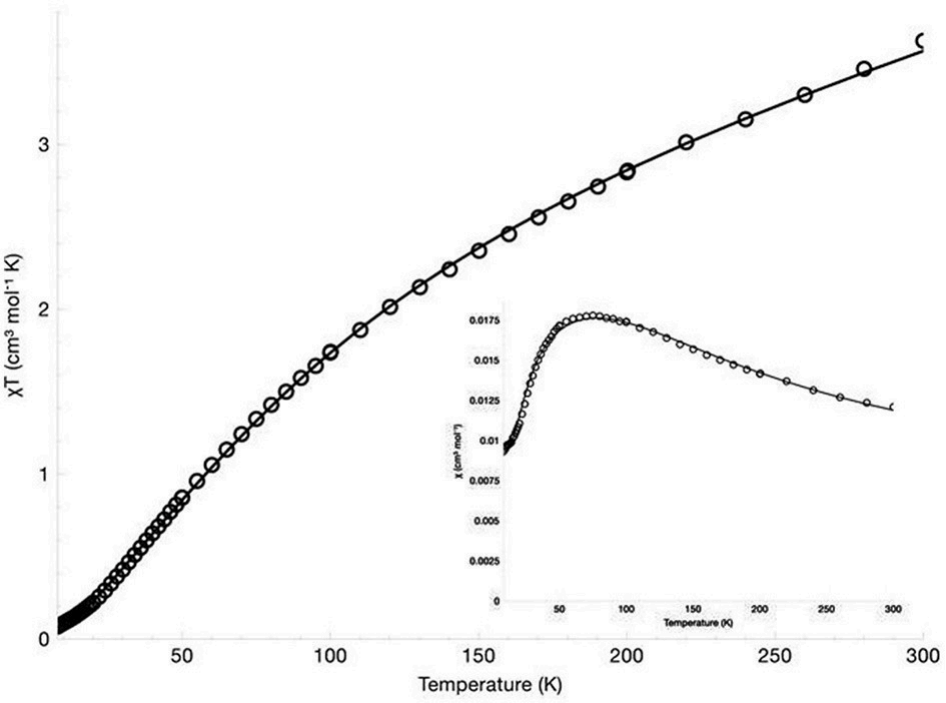
χ_*M*_ vs. T, and χ_*M*_T vs. T (insert) plots for [Ni_2_(L1)(μ-OAc)(H_2_O)_2_](ClO_4_)_2_·H_2_O.

**Figure 5:**
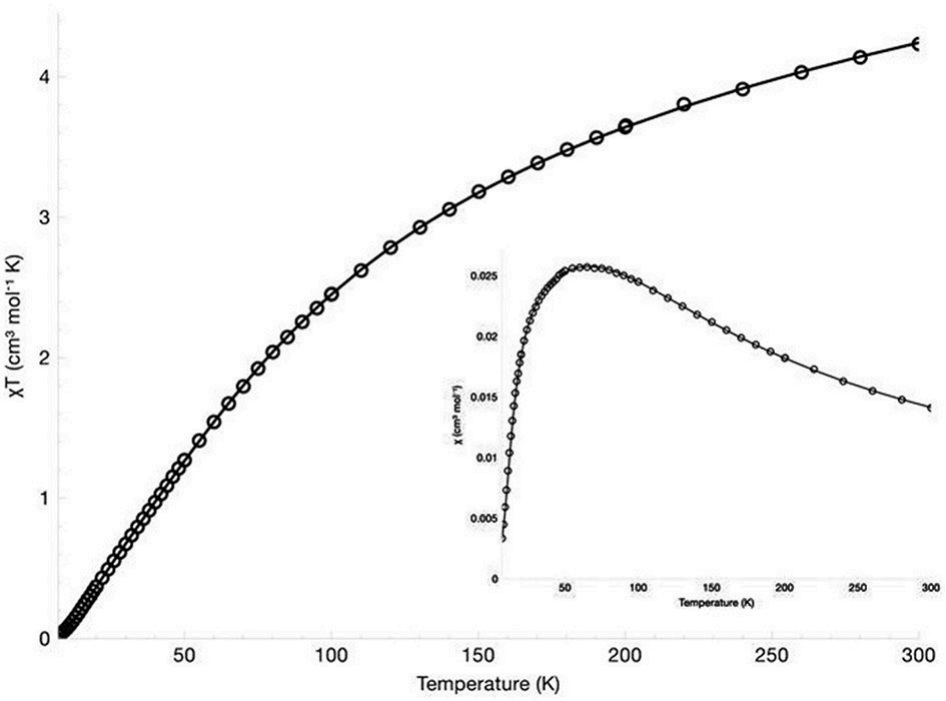
χ_*M*_ vs. T, and χ_*M*_T vs. T (insert) plots for [Co_2_(L1)(μ-OAc)](ClO_4_)_2_·0.5H_2_O.

The magnetic susceptibility of the diNi(II) and diCo(II) complexes was measured over the temperature range 300 to 7 K in an applied field of 500 G. The χ_*M*_ vs. T, and χ_*M*_T vs. T plots are presented in Figure 4 for [Ni_2_(L1)(μ-OAc)(H_2_O)_2_](ClO_4_)_2_·H_2_O and Figure 5 for [Co_2_(L1)(μ-OAc)](ClO_4_)_2_·0.5H_2_O. For both complexes the susceptibility data were fitted using the program PHI (Chilton et al., 2013) with the isotropic exchange Hamiltonian $\hat{\text{H}}$ = −2*J*${\hat{\text{S}}}_{1}\cdot {\hat{\text{S}}}_{2}$.

For [Ni_2_(L1)(μ-OAc)(H_2_O)_2_](ClO_4_)_2_·H_2_O the χ_*M*_T vs. T plot indicates antiferromagnetic coupling between the two Ni(II) centers, with χ_*M*_T gradually decreasing from 3.63 cm^3^ K mol^−1^ (μ_eff_ = 5.39 μ_B_) at 300 K to 0.075 cm^3^ K mol^−1^ (μ_eff_ = 0.77 μ_B_) at 8 K. The room temperature value of χ_M_T is larger than the spin-only value for two non-interacting high-spin nickel(II) ions (χ_*M*_T = 2.00 cm^3^ K mol^−1^, μ_SO_ = 4.00 μ_B_, *g* = 2.00, S = 1). While no orbital angular momentum is expected for the ^3^A_2g_ ground state of the octahedral d^8^ centers, it is expected for the excited ^3^T_2g_ and ^3^T_1g_ states. The theoretical χ_*M*_T value with orbital angular momentum included is 4.99 cm^3^ K mol^−1^ (μ_SL_ = 6.32 μ_B_, L = 3), which suggests some orbital contributions are present in this case. The best fit gave parameters *J* = −27.4 cm^−1^, *g* = 2.29, *D* = 28.4 cm^−1^ and χ_TIP_ = 4.75 × 10^−9^ m^3^ mol^−1^. The inclusion of neither a rhombic zero-field splitting (ZFS) parameter E nor an intermolecular magnetic exchange parameter *zJ* led to an improvement of the fit and were thus omitted.

For [Co_2_(L1)(μ-OAc)](ClO_4_)_2_·0.5H_2_O the magnetic moment has been reported as 4.09 μ_*B*_/Co(II), measured in d_3_-acetonitrile at room temperature (Siluvai and Murthy, 2009). In the study reported herein, the variable temperature magnetic susceptibility of the complex was measured from 300–7 K. The χ_*M*_T value at 300 K is 4.23 cm^3^ K mol^−1^ (μ_eff_ = 5.82 μ_B_), which is within the range of similar binuclear cobalt(II) complexes (Zeng et al., 2004; Tian et al., 2007, 2008; Massoud et al., 2008; Jung et al., 2009; Daumann et al., 2013a; Khandar et al., 2015; Li et al., 2015; Alam et al., 2016). The value is higher than the expected spin-only value for two non-interacting high-spin cobalt(II) ions (3.74 cm^3^ K mol^−1^, μ_SO_ = 5.47 μ_B_, *g* = 2.00, S = 3/2) but significantly lower than expected with the inclusion of orbital angular momentum (6.76 cm^3^ K mol^−1^, μ_SL_ = 7.35 μ_B_, L = 3), suggesting only very minor orbital contributions. The value of χ_*M*_T shows a gradual decrease with decreasing temperature and reaches 0.025 cm^3^ K mol^−1^ at 7 K, indicative of antiferromagnetic coupling between the two centers. The best fit to the data gave parameters *J* = −14.9 cm^−1^, *g* = 2.16 and TIP = 2.22 × 10^−9^ m^3^ mol^−1^. The inclusion of intermolecular magnetic exchange *zJ* was found to have little effect on the fit and, therefore, was not included. The fitted *g* value is larger than the free ion *g* value (*g*_*e*_ = 2.00) and is explained by second-order effects. While the ^4^A_2_′ ground state arising from the trigonal bipyramidal coordination of a d^7^ ion has no orbital angular momentum, admixture of the excited ^4^E″ state with the orbital angular momentum introduces second-order orbital momentum, resulting in a larger *g* value and magnetic moment (Hempel and Miller, 1981; Hossain and Sakiyama, 2002; Bai et al., 2005).

A computational study was undertaken employing the B3LYP functional, Noodleman's broken symmetry, the TZV basis set and the Orca set of programs (Noodleman et al., 1988; Schaefer et al., 1992; Becke, 1993; Neese, 2012; Frisch et al., 2013) in order to calculate the magnitude of the coupling in both complexes (Comba et al., 2009). The calculations were performed based on the X-ray structural parameters for the respective complexes. For the [Ni_2_(L1)(μ-OAc)(H_2_O)_2_](ClO_4_)_2_·H_2_O complex the computed value of *2J* was similar to the experimentally determined value (−26.4 cm^−1^ compared with −27.4 cm^−1^, respectively). For [Co_2_(L1)(μ-OAc)](ClO_4_)_2_·0.5H_2_O the difference in the calculated and experimental values was greater (−20.1 cm^−1^ compared with −14.9 cm^−1^, respectively).

Attempts have been made to correlate structural parameters with the strength of coupling for both diNi(II) and diCo(II) complexes (Nanda et al., 1994a,b,c; Schultz et al., 1997; Johansson et al., 2008; Tomkowicz et al., 2012; Daumann et al., 2013b). In the case of diNi(II) complexes, an initial study reported a linear correlation between the magnitude and sign of *J* with the Ni-O-Ni angle for five diNi(II) complexes of the macrocyclic ligand 1^5^,9^5^-dimethyl-3,7,11,15-tetraaza-1,9(1,3)-dibenzenacyclohexadecaphane-1^2^,9^2^-diol, with aqua, thiocyanate, methanol, imidazole, and pyridine ligands completing the coordination sphere (Nanda et al., 1994c). The authors proposed that at an Ni-O-Ni angle of 97° a cross over from antiferro- to ferro-magnetic coupling occurred (Nanda et al., 1994c). The study was subsequently expanded to include examples of other diNi(II) complexes with phenoxido-bridged ligands and it was concluded that, with the availability of more structural data, a more definitive correlation between *J* and the Ni-O-Ni angle could be expected (Nanda et al., 1994a,b). In line with the previous analysis of the relationship between *J* and Ni-X bonds lengths it was suggested that, in hexacoordinate complexes, the antiferromagnetic coupling was amplified with an increase in bond lengths of one of the axial bonds (Nanda et al., 1994a). Further, the studies suggested that a significant increase in the magnitude of *-J* occurred in the situation where a tetragonally elongated hexacoordinate complex transformed to a five-coordinate square pyramidal complex (Nanda et al., 1994a). Subsequently, a series of studies reported and expanded on the correlations between the sign and magnitude of *J* and both the Ni-O-Ni and Ni-X bond lengths (Allen et al., 1978; Wages et al., 1993; Halcrow and Christou, 1994; Adams et al., 2001; Bu et al., 2001; Mochizuki et al., 2004; Prushan et al., 2007; Greatti et al., 2008; Pawlak et al., 2008; Mandal et al., 2009; Chattopadhyay et al., 2010; Ren et al., 2011; Biswas et al., 2012, 2017; Botana et al., 2014; Mahapatra et al., 2016; Massoud et al., 2016; Sanyal et al., 2016; Xavier and Neves, 2016). As the number of studies, and hence the number of examples of diNi(II) complexes with various bridging ligand types, has increased the relationship between *J* and a structural parameter (Ni-O-Ni angle; Ni-X distance) has been described in terms of both linear, albeit with considerable scatter (Mahapatra et al., 2016), and polynomial functions (Bu et al., 2001). Clearly, the relationship is dependent on a set of parameters and even on the type of bridging ligands (Krupskaya et al., 2010; Botana et al., 2014). The sign and magnitude of *J* (−27.4 cm^−1^) for [Ni_2_(L1)(μ-OAc)(H_2_O)_2_](ClO_4_)_2_·H_2_O are consistent with the Ni^II^-μO-Ni^II^ angle of 131.6(2)° (Table S1 and Figure S1) (Nanda et al., 1994c; Mochizuki et al., 2004; Biswas et al., 2012, 2017; Massoud et al., 2016).

The relationship between the structural parameters and the magnitude of the observed magnetic coupling for dicobalt(II) complexes has been reviewed (Arora et al., 2012, p. 703; Tomkowicz et al., 2012; Daumann et al., 2013a). It was concluded that for complexes with a μ-O_bridge_/bis(μ_2_-RCOO-κ^2^O:O') core, the variations in magnitude of *J* could be related to the type of μ-O_bridge_, the Co-O_bridge_-Co angle and the type of R-group (Tomkowicz et al., 2012). Further, it was proposed that the strength of the coupling varied according to bridge type (μ-O^2−^>μ-OH^−^>μ-H_2_O) (Schultz et al., 1997), and that Co(II)-O-Co(II) bond angles around 96° in some examples resulted in ferromagnetic coupling via orthogonal magnetic orbitals (Tudor et al., 2008; Fabelo et al., 2009; Tomkowicz et al., 2012), and it was also suggested that bis(μ_2_-*syn,syn*-CH_3_COO-κ^2^*O:O'*) bond angles were important (Arora et al., 2012). Extremely weak antiferro- or ferro-magnetic coupling was proposed for diCo(II) complexes with the μ-O_(phenoxido)_;bis(μ_2_-OAc-κ^2^*O:O'*) bridge, although exceptions occurred (Arora et al., 2012; Daumann et al., 2013a). Complexes with the μ-H_2_O;bis(μ_2_-RCOO-κ^2^*O:O'*) core appear to promote weak antiferromagnetic coupling, although stronger than that seen with the μ-O_(phenoxido)_ analog; the bis(μ_2_-RCOO-κ^2^*O:O'*);μ_2_-O;κ^2^*O,O'*-CH_3_COO core appeared to promote ferromagnetic coupling (Daumann et al., 2013a). The relationship between Co(II)-X bond distances and the magnitude and sign of *J* was extremely weak, and both the Co(II)^.^Co(II) distance and extent of distortion around the Co(II) center seemingly having little bearing on the coupling (Daumann et al., 2013a). Of the structural parameters considered in this analysis, the Co(II)-X-Co(II) bridge angles appear to have some influence on the sign and magnitude of *J* (Daumann et al., 2013a), in agreement with earlier studies, but none of the structural relationships appears to be particularly strong (Table S2 and Figure S2; Johansson et al., 2008; Tudor et al., 2008; Fabelo et al., 2009; Tomkowicz et al., 2012).

### Phosphoesterase activity

**Figure 6:**
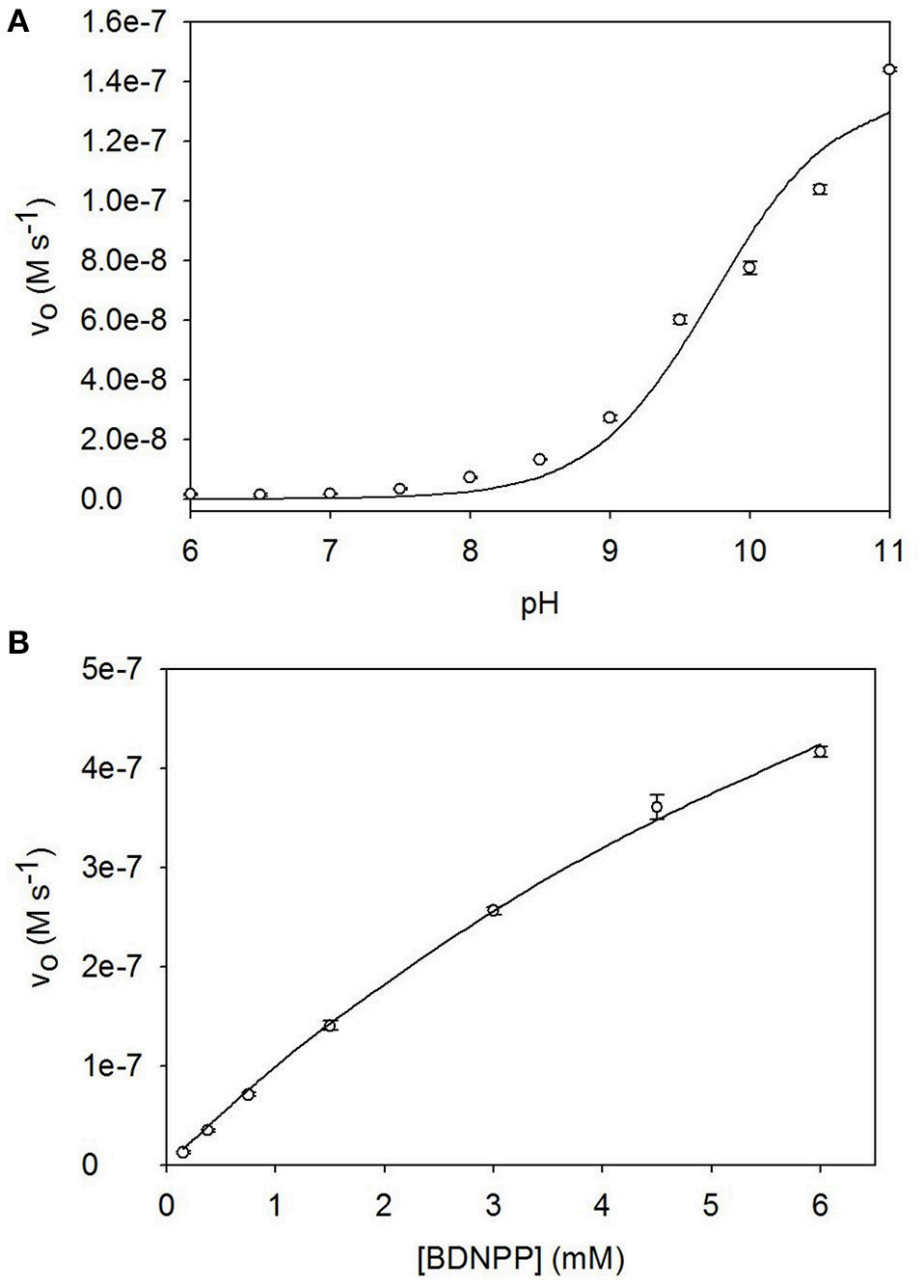
**(A)** pH dependence profile, and **(B)** Michaelis-Menten plot for the hydrolysis of BDNPP catalyzed by [Ni_2_(L1)(μ-OAc)(H_2_O)_2_](ClO_4_)_2_·H_2_O.

The pH dependence of BDNPP hydrolysis by [Ni_2_(L1)(μ-OAc)(H_2_O)_2_](ClO_4_)_2_·H_2_O was analyzed between pH 4.75 and pH 11.0; the rate enhances sharply at pH values ≥9.0, reaching a maximum at pH ≥11.0. Data were fit as described in the Experimental section and resulted in an estimate of the p*K*_a_ value (9.7 ± 0.1) of the catalytically relevant protonation equilibrium (Figure 6A). Since the measurements of the catalytic rates as a function of [S] did not reach full saturation obtained catalytic parameters need to be viewed with some caution. A combination of non-linear and linear regression analyses indicate that plausible values for V_max_ and *K*_*m*_ are around 6–7 × 10^−5^ M.min^−1^ and 10 mM, respectively (Figure 6B). For the following comparison of the catalytic efficiencies of a number of Ni-dependent biomimetics we will use the rate of hydrolysis of 6 mM [S] by 40 μM [Ni_2_(L1)(OAc)(H_2_O)_2_](ClO_4_)_2_·H_2_O, i.e., 2.50 × 10^−5^ M.min^−1^. This rate corresponds to a *k*_*cat*_ of ~0.01 s^−1^.

**Scheme 1:**
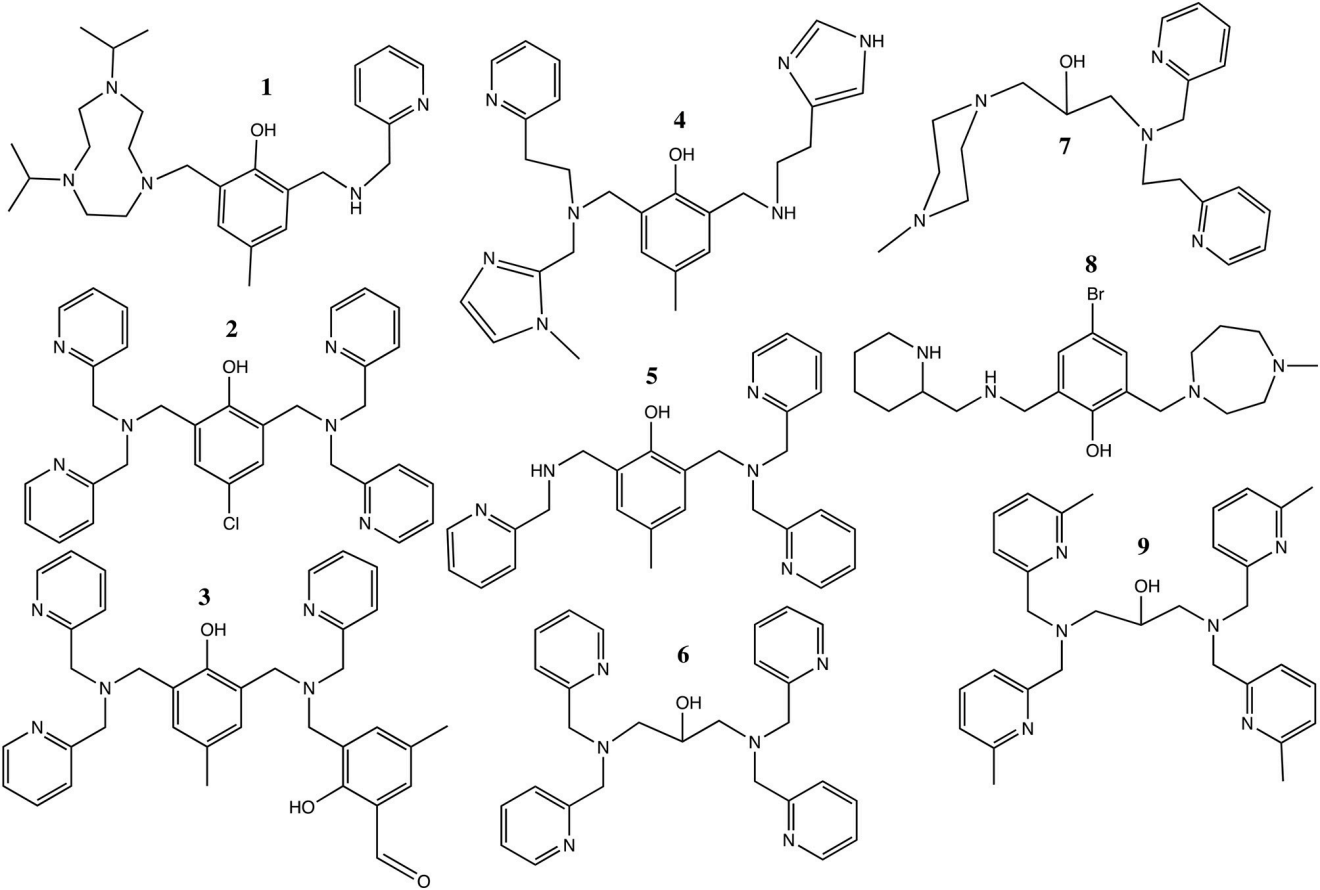
The ligands listed in Table 3.

Several previous studies focused on the phosphatase-like activity of di-Ni(II) complexes (Table 3 and Scheme 1; Yamaguchi et al., 1997, 2001; Yamane et al., 1997, 2001; Parimala and Kandaswamy, 2003; Jikido et al., 2005; Greatti et al., 2008; Ren et al., 2011; Piovezan et al., 2012; Wu and Wang, 2014; Massoud et al., 2016; Xavier and Neves, 2016). In a number of cases the experimental conditions in terms of solvent (acetonitrile/aqueous buffer) and substrate (BDNPP) were similar to those employed in the present work, although the pH at which the *k*_*cat*_ and *K*_*m*_ values were determined did vary, making direct comparisons difficult (Greatti et al., 2004, 2008; Piovezan et al., 2012; Massoud et al., 2016; Xavier and Neves, 2016). The nucleophilic agent in these reactions has been proposed as either a terminal (Yamaguchi et al., 2001; Vichard and Kaden, 2002; Parimala and Kandaswamy, 2003; Jikido et al., 2005; Greatti et al., 2008; Massoud et al., 2016; Sanyal et al., 2016) or a bridging (Ren et al., 2011; Piovezan et al., 2012; Wu and Wang, 2014) hydroxido moiety.

**Table 3:** Michaelis Menten kinetic data of dinuclear Ni(II) complexes that mimic metallophosphatases.

**Ligand^a^**	**Complex**	**Substrate**	**Solvent**	**k_cat_ (s^−1^)**	**K_m_ (mM L^−1^)**	**References**
2-[(4,7-diisopropyl-1,4,7-triazonan-1-yl)methyl]-4-methyl-6-[(pyridine-2-ylmethylamino)methyl]phenol (HL) (**1**)	[Ni_2_(L)(μ-OAc)_2_(OH_2_)](BPh_4_).H_2_O	BDNPP	Acetonitrile/aqueous buffer	0.013 (pH 9)	3.44	Xavier and Neves, 2016
2,6-bis[bis(2-pyridylmethyl)aminomethyl]-4-chlorophenol (HL^Cl^O) (**2**)	[Ni_2_(L^Cl^O)(μ-OAc)_2_](PF_6_).3H_2_O	BDNPP	Acetonitrile/aqueous buffer	2.80 × 10^−3^ (pH 7) 0.065 (pH 10.5)	0.21 (pH 7) 2.18 (pH 10.5)	Massoud et al., 2016
2-[(N-benzyl-N-2-pyridylmethylamine)]-4-methyl-6-[N-(2-pyridylmethyl)aminomethyl)])-4-methyl-6-formylphenol) (H_2_BPPAMFF) (**3**)	[Ni_2_(HBPPAMFF)(μ-OAc)_2_(H_2_O)](BPh_4_)	BDNPP	Acetonitrile/aqueous buffer	0.054 (pH 9)	1.57	Piovezan et al., 2012
2-[N-(2-(pyridyl-2-yl)ethyl)(1-methylimidazol-2-yl)aminomethyl]-4-methyl-6-[N-(2-(imidazol-4-yl)ethyl)aminomethyl]phenol (HL2) (**4**)	[Ni_2_(L2) (μ-OAc)_2_(CH_3_CN)](BPh_4_)	BDNPP	Acetonitrile/aqueous buffer	0.034 (pH 9)	1.19	Greatti et al., 2008
2-[N-bis-(2-pyridylmethyl)aminomethyl]-4-methyl-6-[N-(2-pyridylmethyl)aminomethyl] phenol (HL_A_1) (**5**)	[Ni_2_(L_A_1) (μ-OAc)_2_(H_2_O)]ClO_4_.H_2_O	BDNPP	Acetonitrile/aqueous buffer	0.386 (pH 9)	5.67	Greatti et al., 2004, 2008
1,3-bis(bis(pyridin-2-ylmethyl)amino)propan-2-ol (HL^1^) **(6)**	[Ni_2_L^1^(μ-OAc)(H_2_O)_2_](ClO_4_)_2_·H_2_O	BDNPP	Acetonitrile/aqueous buffer	~0.025 (pH 11)	10	This work
N-4-methyl-homopiperazine-N'-[N-(2-pyridylmethyl)-N-2-(2-pyridylethylamine]-1,3-diaminopropan-2-ol (HL) (**7**)	[Ni_2_(L)(OH)]^2+^	BNPP	Aqueous buffer	7.4 × 10^−5^ (pH 8.4)	6.95	Wu and Wang, 2014
2-[[(2-piperidylmethyl)amino]methyl]-4-bromo-6-[(1-methylhomopiperazine-4-yl)methyl]phenol (HL) (**8**)	[Ni_2_(L)(OH)]^2+^	BNPP	Ethanol/aqueous buffer	1.49 × 10^−4^ (pH 8.3)	7.25	Ren et al., 2011
N,N,N',N'-tetrakis[(6-methyl-2-pyridyl)methyl]-1,3-diaminopropan-2-ol (Me_4_tpdpH) (**9**)	[Ni_2_(Me_4_tpdp) (μ-OAc)_2_(H_2_O)] (ClO_4_)	BNP	Acetonitrile/aqueous buffer	k_BNP_ = 3.4 × 10^−2^ M^−1^ s^−1^		Yamaguchi et al., 2001

a *The numbers refer to the ligands shown in Scheme 1*.

Of the examples listed in Table 3, the complex with the ligand 2-[N-bis-(2-pyridylmethyl)aminomethyl]-4-methyl-6-[N-(2-pyridylmethyl)aminomethyl]phenol, [Ni_2_(L_A_1) (μ-OAc)_2_(H_2_O)]ClO_4_.H_2_O, was the most effective and efficient catalyst for the hydrolysis of BDNPP with *k*_*cat*_ = 0.386 s^−1^ at pH 9 (Greatti et al., 2008). The rate enhancement was rationalized in terms of the fact that in solution at pH 9.00, the active species [Ni_2_(L_A_1)(H_2_O)_2_(μ-OH)]^2+^ made up approximately 85% of the complexes present in solution (Greatti et al., 2008). The proposed mechanism for the reaction involved initial loss of the bridging acetate ligands and coordination of BDNPP after replacement of one aqua ligand (Greatti et al., 2008). The substrate is thus proposed to coordinate in a monodentate manner and orient *cis* to a terminal Ni-OH moiety, the latter promoting a nucleophilic attack on the phosphorus atom with release of the nitrophenolate anion and formation of a bridging DNP molecule. A subsequent intramolecular nucleophilic attack by a μ-OH moiety on the μ-DNP was proposed to result in loss of a second nitrophenolate anion and formation of coordinated phosphate anion which was subsequently replaced by H_2_O/OH^−^ to complete the cycle (Greatti et al., 2008). In the case of the complex [Ni_2_(Me_4_tpdp)(μ-OAc)_2_(H_2_O)](ClO_4_), a mechanism was proposed whereby the substrate, bis(4-nitrophenyl)phosphate (BNP) was coordinated in a μ-1,3 manner to the diNi(II) complex, after loss of the acetate ligands, with subsequent nucleophilic attack by a terminal Ni-OH moiety at the phosphorus center (Yamaguchi et al., 2001). In that case the reaction is facilitated by the fact that the relevant hydroxido ligand is arranged *cis*, and thus adjacent, to the substrate (Yamaguchi et al., 2001). A variation of the above mechanisms was proposed for [Ni_2_(L^Cl^O)(μ-OAc)_2_](PF_6_).3H_2_O by Massoud et al.; in the initial phase of the catalytic cycle one of two metal-bridging acetate groups is displaced and the substrate BDNPP binds to one of the Ni(II). Subsequently, dependent on pH, the attack of a terminal hydroxide that either resides on the same (high pH) or the opposite (low pH) metal as the substrate attacks the phosphorus moiety, leading to the DNPP product being coordinated either bidentately to one metal or to both metals, respectively (Massoud et al., 2016).

**Figure 7:**
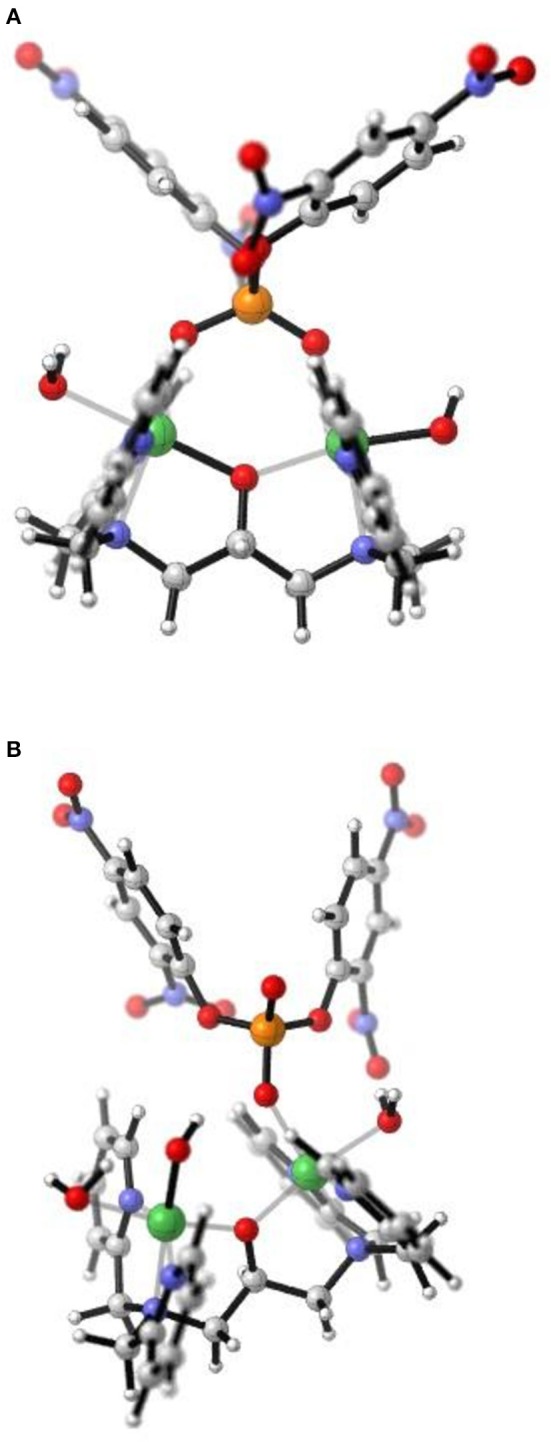
After loss of the μ-acetato ligands in the complex [Ni_2_(L1)(μ-OAc)(H_2_O)_2_] ^2+^ these structures represent **(A)** μ-1,3 coordination of BDNPP^−^ to the two Ni(II) sites with two aqua ligands positioned *trans* to the μ-alkoxo moiety; and **(B)** monodentate coordination of BDNPP- with a OH/H_2_O ligand occupying the vacant site on the second Ni(II) and poised as the intramolecular terminal nucleophile. Structures generated from DFT calculations.

**Figure 8:**
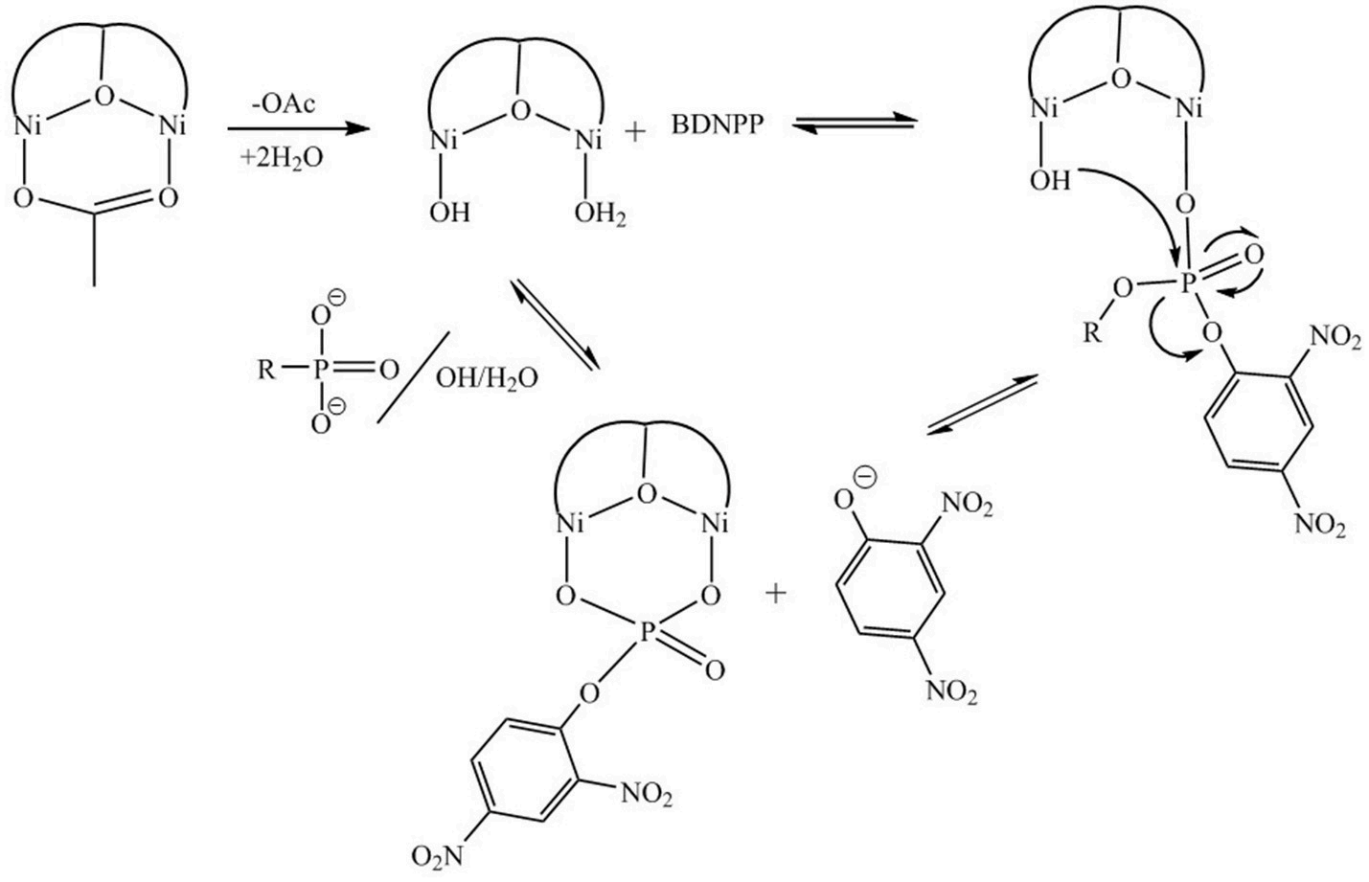
Proposed mechanism of reaction between [Ni_2_(L1)(μ-OAc)(H_2_O)_2_]^2+^ and BDNPP^−^ adapted from (Vichard and Kaden, 2004). The proposed mechanism resembles that of the lower pH mechanism proposed by Massoud et al. (2016).

For [Ni_2_(L1)(μ-OAc)(H_2_O)_2_](ClO_4_)_2_.H_2_O both Ni(II) sites are six-coordinate. Loss of the μ-acetato ligand is thus a prerequisite for catalytic activity as it generates vacant positions for the substrate to bind (Figure 7A). However, according to the crystal structure (Figure 2) none of the available aqua/hydroxido ligands are positioned suitably to act as nucleophiles for the reaction. We thus propose that the release of the μ-acetato group enables the monodentate coordination of the substrate to one of the Ni(II) ions and a water molecule (with a p*K*_*a*_ of ~9.7) to the other metal ion (Figure 7B). The subsequent attack by the deprotonated water ligand on the phosphorus moiety of the substrate triggers catalysis (Figure 8). Insofar, the model resembles that of the lower pH mechanism proposed by Massoud et al. (2016). In the latter complex the presence of two metal-bridging acetate groups provides the basis for an enhanced mechanistic flexibility (i.e., low and high pH pathways), where the nucleophile can be either bound to the same or the opposite metal ion as the substrate.

The phosphatase-like activity of the analogous [Co_2_L1(μ-OAc)](ClO_4_)_2_·0.5.H_2_O could not be investigated under corresponding experimental conditions since the initially red colored solution turned yellow after the addition of the aqueous buffer solution. The absorbance of the mixture at 400 nm increased upon standing, making any attempt to measure the formation of the dinitrophenolate anion difficult. The change of color of the solution may be explained by the oxidation of cobalt(II) ions (Suzuki et al., 1990).

## Conclusions

The dinickel(II) and dicobalt(II) complexes of 1,3-bis(bis(pyridin-2-ylmethyl)amino)propan-2-ol have been prepared and some of their properties were compared. Magnetic susceptibility studies confirmed that the two metal ions in both complexes are antiferromagnetically coupled and computational studies verified the experimental magnetic coupling constants. Attempted correlation of the relationship between structural parameters, particularly the M-O-M angles, with the strength of the magnetic coupling were only partially successful. Kinetic analysis with the activated substrate BDNPP suggested that for the diNi(II) complex a terminal water is the nucleophile with a kinetically relevant p*K*_*a*_ of 9.7 ± 0.1 and a *k*_*cat*_ value as high as 0.025 s^−1^ (Table 3). The complex is thus at the higher end of the range for catalytic efficiency for similar diNi(II) complexes with this substrate (the corresponding diCo(II) complex was found to oxidize readily in the buffer solution). Thus, although no suitable nucleophile (OH^−^) is present in the original molecule the replacement of the two acetate bridges by water and/or substrate molecules (Figure 8) may not be rate-limiting. The complex is proposed to employ a similar mechanism as proposed for a series of analogous model systems for enzymes such as PAPs (Smith et al., 2009; Comba et al., 2012a,b; Bernhardt et al., 2015; Roberts et al., 2015; Bosch et al., 2016). The majority of complexes listed in Table 3 attain optimal catalytic efficiency under alkaline conditions (>pH 9.0), somewhat higher than the pH optimum of the di-Ni(II) enzyme urease (pH 7.4). This difference may be due to the fact that the majority of model systems use a terminally bound nucleophile to initiate the hydrolytic reaction, whereas urease employs a metal ion-bridging hydroxide (Zambelli et al., 2011). Nonetheless, it is apparent that the complexes listed in Table 3 represent suitable functional models for biological catalysts such as ureases and PAPs.

## Author contributions

AH designed the project and supervised the synthetic chemistry and hydrolytic experiments, contributed to the writing of the manuscript. DE undertook the syntheses of the ligand and the metal complex and undertook the spectroscopic characterization. AR undertook the magnetochemical study and analyzed the results. PC in association with AR supervised the magnetochemical study and data analysis. GS contributed to the design of the project, supplied the funding for the work, and contributed to the writing of the manuscript. EK contributed to the writing of the DFT section of the manuscript and assisted with DFT calculations. LG undertook the X-ray structure analysis, contributed to the analysis of the kinetic data, undertook the DFT studies, and contributed to the writing of the manuscript.

### Conflict of interest statement

The authors declare that the research was conducted in the absence of any commercial or financial relationships that could be construed as a potential conflict of interest.
