# Supplementary material for: Synthesis, Magnetic Properties, and Catalytic Properties of a Nickel(II)-Dependent Biomimetic of Metallohydrolases
Source: Front Chem. 2018 Sep 25;6:441. doi: 10.3389/fchem.2018.00441 (PMC6168013; doi:10.3389/fchem.2018.00441)
Supplement: Supplementary file 1 [file Data_Sheet_1.DOCX]

Supplementary Material for the paper

**Synthesis, Magnetic Properties and Phosphoesterase Activity of Dinuclear Nickel(II) and Cobalt(II) Complexes**

Adolfo Horn, Jr,^a*^ Daniel Englert,^b,c^ Asha E. Roberts,^b^ Peter Comba,^b^ Gerhard Schenk,^c^ Elizabeth H. Krenske,^c^ Lawrence R. Gahan,^c*^

*a) Laboratório de Ciências Químicas, Universidade Estadual do Norte Fluminense, 28013-602, Campos dos Goytacazes/RJ, Brazil*

*b) Anorganisch-Chemisches Institut and Interdisciplinary Center of Scientific Computing, Universität Heidelberg, INF 270, D-69120, Heidelberg, Germany.*

*c) School of Chemistry and Molecular Biosciences, The University of Queensland, Brisbane*

*4072, Queensland, Australia.*

Table S1. Typical values of J (cm^-1^) and Ni-O-Ni bridging angle (°) for diNi(II) complexes.

| Ligand | Complex | Ni-O-Ni (°) | J (cm^-1^) | ref |
| --- | --- | --- | --- | --- |
| 1^5^,9^5^-dimethyl-3,7,11,15-tetraaza-1,9(1,3)-dibenzenacyclohexadecaphane-1^2^,9^2^-diol (H_2_L) | [Ni_2_(L)(H_2_O)_4_](ClO_4_)_2_.4NH_2_CONH_2_ | 99.5 | -17.02 | [[1](#_ENREF_1)] |
| 1^5^,9^5^-dimethyl-3,7,11,15-tetraaza-1,9(1,3)-dibenzenacyclohexadecaphane-1^2^,9^2^-diol (H_2_L) | [Ni_2_(L)(NCS)_2_(H_2_O)_2_].2(CH_3_)_2_NCHO | 99.2 | -21.3 | [[1](#_ENREF_1), [2](#_ENREF_2)] |
| 1^5^,9^5^-dimethyl-3,7,11,15-tetraaza-1,9(1,3)-dibenzenacyclohexadecaphane-1^2^,9^2^-diol (H_2_L) | [Ni_2_(L)(CH_3_OH)_2_(ClO_4_)_2_].2NH(C_2_H_5_)_3_ClO_4_ | 101.3 | -29.5 | [[1](#_ENREF_1)] |
| 1^5^,9^5^-dimethyl-3,7,11,15-tetraaza-1,9(1,3)-dibenzenacyclohexadecaphane-1^2^,9^2^-diol (H_2_L) | [Ni_2_(L)(imidazole)_2_](ClO_4_)_2_ | 104.1 | -49.8 | [[1](#_ENREF_1)] |
| 1^5^,9^5^-dimethyl-3,7,11,15-tetraaza-1,9(1,3)-dibenzenacyclohexadecaphane-1^2^,9^2^-diol (H_2_L) | [Ni_2_(L)(pyridine)_2_](ClO_4_)_2_ | 105.7 | -67.1 | [[1](#_ENREF_1)] |
| 2-[(2-dimethylamino-ethylamino)-  methyl]-phenol (HL) | [Ni_2_L_2_(NO_2_)_2_] | 101.77 | -39 | [[3](#_ENREF_3)] |
| 2-[(2-dimethylamino-ethylamino)-  methyl]-phenol (HL) | [Ni_3_L_3_(OH)(NO_2_)](ClO_4_) | 86.66-96.07 | +18.2 | [[3](#_ENREF_3)] |
| 7,7'-(2-hydroxypropane-1,3-diyl)bis[3,7,11,17-tetraazabicyclo[11.3.1]-  heptadeca-1(17),13,15-triene] (HL) | [Ni_2_(L)N_3_](N_3_)_2_.7H_2_O | 140.08 | -29.7 | [[4](#_ENREF_4)] |
| 2-  [1-(3-methylamino-propylamino)-ethyl]-phenol (HL1) | [Ni_2_(L1)_2_(NCS)_2_] | 104.54 | -23.32 | [[5](#_ENREF_5)] |
| 2-[1-  (2-dimethylamino-ethylamino)-ethyl]-phenol (HL2), | Ni_2_(L2)_2_(NCS)_2_] | 102.92 | -35.45 | [[5](#_ENREF_5)] |
| 2-[1-  (3-dimethylamino-propylamino)-ethyl]-phenol (HL3) | Ni_2_(L3)_2_(NCS)_2_] | 103.84 | -34.02 | [[5](#_ENREF_5)] |
| 1^5^,9^5^-dimethyl-3,7,11,15-tetraaza-1,9(1,3)-dibenzenacyclohexadecaphane-1^2^,9^2^-diol (H_2_L) | [Ni_2_(L)(µ-OAc)(H_2_O)_2_](ClO_4_).CH_3_OH.H_2_O | 95.1  90.8 | 2.94 | [[6](#_ENREF_6)] |
| 2-[[(2-piperidylmethyl)amino]-  methyl]-4-bromo-6-[(1-methylhomopiperazine-4-yl)methyl]phenol (HL) | [Ni_2_(L)(µ-DNBA)_2_(CH_3_CN)_2_](BPh_4_) | 119.5 | -1.8 | [[7](#_ENREF_7)] |
| 2[[(2-piperidylmethyl)amino]-  Methyl]-4-bromo-6-[(1-methylhomopiperazine-4-yl)methyl]phenol (HL) | [Ni_2_(L)(µ-BPP)_2_(CH_3_CN)_2_]-  (BPh_4_) | 126.1 | -7.7 | [[7](#_ENREF_7)] |
| 2,6-bis[bis(2-pyridylmethyl)  aminomethyl]-4-chlorophenol (L^Cl^OH) | [Ni_2_(μ-L^Cl^O)(μ_2_-OAc)_2_](PF_6_)·3H_2_O | 115.70 | -3.70 | [[8](#_ENREF_8)] |
| 2,6-bis[bis(2-pyridylmethyl)  aminomethyl]-4-chlorophenol (L^Cl^OH) | [Ni_2_(μ-L^Cl^O)(μ_2_-OAc)_2_](ClO_4_)·CH_3_COCH_3_ | 116.43 | -1.01  (D=-4.96)  -1.20  (D=+2.8) | [[8](#_ENREF_8)] |
| 1,4,8-triazacycloundecane (tacud) | [Ni_2_(tacud)_2_(µ-H_2_O)(µ-Cl)_2_]Cl_2_ | 90.49 | +2.28 | [[9](#_ENREF_9)] |
| 2-(((2-(1H-imidazol-4-yl)ethyl)amino)methyl)-4-methyl-6-((((1-methyl-1H-imidazol-2-yl)methyl)(2-(pyridin-2-yl)ethyl)amino)methyl)phenol (HL2 ) | [Ni_2_(L2)(µ-OAc)_2_(CH_3_CN)](BPh_4_) | 117.78 | -3.4 | [[10](#_ENREF_10)] |
| 2,6-bis[[bis(2-pyridylmethyl)amino]methyl]-4-methylphenol (H2L) | [Ni_2_(L**)**(µ-OAc)_2_](BF_4_)·2MeOH | 114.5 | -2.3 | [[11](#_ENREF_11)] |
| 2,6-bis(N-ethylpiperazine-iminomethyl)-4-methylphenol (LH) | [Ni_2_(LH_2_)(H_2_O)_2_(OH)(NO_3_)](NO_3_)_3_ | 93.26 (µ-phenoxide)  96.2 (µ-OH) | 44.26 | [[12](#_ENREF_12)] |
| 2,6-bis[N-methyl-N-(2-pyridylethyl)amino]-4-methylphenol (HL1) | [Ni_2_(L1)(µ-OAc)_2_(H_2_O)_2_](PF_6_).MeOH. 3H_2_O | 126.19 | -48.4* | [[13](#_ENREF_13)] |
| N^1^,N^1^,N^2^,N^2^-tetramethylethane-1,2-diamine (tmen) | [Ni_2_(OAc)_3_(urea)(tmen)_2_](OTf) | 109.76 | -0.9 | [[14](#_ENREF_14)] |
| 2,6-bis[(bis((1-methylimidazol-2-yl)methyl)-  amino)methyl]-4-methylphenol (Hbimp) | [Ni_2_(bimp)(µ-OAc)_2_](ClO_4_).MeOH | 116.7 | -1.9 | [[15](#_ENREF_15)] |
| 1,4,7-trimethyl-1,4,7-triazonane (Me_3_tacn) | [Ni_2_(Me_3_tacn)(µ-OH)(µ-OAc)_2_] | 115.2 | -4.5 | [[16](#_ENREF_16)] |
| 1,3-bis(bis(pyridin-2-ylmethyl)amino)propan-2-ol **(**HL^1^**)** | [Ni_2_L^1^(OAc)(H_2_O)_2_](ClO_4_)_2_·H_2_O | 131.6 | -27.4 | This work |

** H = -JS_1_.S_2_*

**Table S2**. Typical values of J (cm^-1^) and Ni-O-Ni bridging angle (°) for diCo(II) complexes [[17-19](#_ENREF_17)]

| Ligands | Complex | J  (cm^–1^) | Bridge angles  (deg) | ref |
| --- | --- | --- | --- | --- |
| *N*,*N*,*N*′,*N*′-tetramethylethylenediamine | [Co_2_(*μ*-CH_3_COO)_3_(urea)(tmen)_2_](OTf) | +18.0^a^ | 107.7 | [[18](#_ENREF_18), [20](#_ENREF_20), [21](#_ENREF_21)] |
| 2-(3,5-dimethyl-1H-pyrazol-1-yl)-4,6-dimethylpyrimidine | [Co_2_(PymPz)_2_Cl_4_] | +12.83^a^ | 94.87 | [[22](#_ENREF_22)] |
| 2-(3,5-dimethyl-1H-pyrazol-1-yl)-4,6-dimethylpyrimidine | [Co_2_(PymPz)_2_(N_3_)_4_] | +10.11^a^ | 102.38 | [[22](#_ENREF_22)] |
| 1,2,4,5-benzenetetracarboxylic acid (H_4_bta) | [Co_2_(bta)(H_2_O)_6_]_n_.2nH_2_O | +5.4^b^ | 93.89  92.16 | [[18](#_ENREF_18), [23](#_ENREF_23)] |
| 2,6-bis[3-(pyridin-2-yl)pyrazol-1-ylmethyl]-4-methylphenol (HL2) | [Co_2_(L^2^)(*μ*-CH_3_COO)_2_(CH_3_CN)_2_](BPh_4_) | +2.51^b^ | 112.83 | [[24](#_ENREF_24)] |
| 1,4-phenylenediacetic acid (H_2_phda) | [Co(phda)(H_2_O)]_n_.nH_2_O | +2.16^b^ | 99.13  93.03 | [[18](#_ENREF_18), [23](#_ENREF_23)] |
| 4-bromo-2,6-bis(((2-methoxyethyl)(pyridin-2-ylmethyl)amino)methyl)phenol (BrHL2) | [Co_2_(BrL2)(*μ*-CH_3_COO)_2_](PF_6_) | +3.09^b^ | 113.48 | [[19](#_ENREF_19)] |
| 2,6-bis(((2-methoxyethyl)(pyridin-2-ylmethyl)amino)methyl)-4-nitrophenol (NO_2_HL2) | [Co_2_(NO_2_L2)(*μ*-CH_3_COO)_2_](PF_6_) | +0.78^b^ | 112.49 | [[19](#_ENREF_19)] |
| ethyl 4-hydroxy-3,5-bis(((2-methoxyethyl)(pyridin-2-ylmethyl)amino)methyl)benzoate (CO_2_EtHL2) | [Co_2_(CO_2_EtL2)(*μ*-CH_3_COO)_2_](PF_6_) | -0.10^b^ | 112.26 | [[19](#_ENREF_19)] |
| 2,6-bis[bis(2-hydroxyethyl) aminomethyl]-4-methylphenol (Hbhmp) | [Co_2_(bhmp)(*μ*-CH_3_COO)_2_](BPh_4_) | -0.37^b^ | 112.2 | [[25](#_ENREF_25), [26](#_ENREF_26)] |
| 2,6-bis[bis(2-methoxyethyl) aminomethyl]-  4-methylphenol (Hbomp) | [Co_2_(bomp)(*μ*-CH_3_COO)_2_](BPh_4_) | -0.46^b^ | 113.02 | [[25](#_ENREF_25), [26](#_ENREF_26)] |
| ethyl 4-hydroxy-3,5-bis(((2-hydroxyethyl)(pyridin-2-yl­methyl)­amino)methyl)benzoate (CO_2_EtH_3_L1) | [Co_2_(CO_2_EtL1)(*μ*-CH_3_COO)_2_](PF_6_) | -0.66^b^ | 114.55 | [[19](#_ENREF_19)] |
| 2,6-bis(((2-methoxyethyl)(pyridin-2-ylmethyl)amino)methyl)-4-methylphenol (CH_3_HL2) | [Co_2_(CH_3_L2)(*μ*-CH_3_COO)_2_](PF_6_) | -0.67^b^ | 112.91 | [[19](#_ENREF_19)] |
|  | [Co_3_(μ-CF_3_COO)_4_(*μ*-H_2_O)_2_(CF_3_COO)_2_ (H_2_O)_2_(C_4_H_8_O_2_)].2C_4_H_8_O_2_ | -0.4^a^ | 115.1 | [[27](#_ENREF_27)] |
| imidazole | [Co_2_(*μ*-CH_3_COO)(O(N)(O=C)_2_(CH_2_)_3_)  (Im)_4_][OTf]_2_ | -1.0^a^ | 126.63 | [[28](#_ENREF_28)] |
| 1,10-phenanthroline | [Co_2_(*μ*-H_2_O)(*μ*-CH_3_COO)_2_(CH_3_COO)_2_(phen)_2_] | -2.1^b^ | 114.01 | [[29](#_ENREF_29)] |
| *N*,*N*,*N*′,*N*′-tetramethylethylenediamine | [Co_2_(*μ*-H_2_O)(*μ*-CH_3_COO)_2_(CH_3_COO)_2_(tmen)_2_] | -1.2^a^ | 115.1 | [[30](#_ENREF_30)] |
| Isopropyl alcohol | [Co_2_(*μ*-OH_2_)(*μ*-CCl_3_COO)_2_(H_2_O)_2_  (CCl_3_COO)_2_(IPA)_2_] | -1.5^a^ | 116.81 | [[18](#_ENREF_18)] |
| dioxane | [Co_2_(*μ*-OH_2_)(*μ*-CCl_3_COO)_2_(H_2_O)_2_  (CCl_3_COO)_2_(DIOX)_2_] | -1.5^a^ | 115.6 | [[18](#_ENREF_18)] |
| HOOC(CH3)3 (PivH) | [Co_2_(*μ*-OH_2_)(*μ*-Piv)_2_(Piv)_2_(HPiv)_4_] | -1.6^a^ | 111.38 | [[18](#_ENREF_18)] |
| imidazole | [Co_2_(Im)_~~4~~_(CH_3_COO)_4_(H_2_O)] | -1.6^a^ | 117.2 | [[17](#_ENREF_17)] |
|  | [Co_2_(*μ*-OH_2_)(*μ*-CCl_3_COO)_2_(H_2_O)_2_ (CCl_3_COO)_2_(THF)_2_] | -2.1^a^ | 115.98 | [[18](#_ENREF_18)] |
|  | [Co_2_(*μ*-OH_2_)(*μ*-CCl_3_COO)_2_(H_2_O)_2_  (CCl_3_COO)_2_(glyme)_2_] | -2.1^a^ | 113.59 | [[18](#_ENREF_18)] |
| 3-[(furan-2-ylmethylimino)methyl]-2-hydroxy-5-  Methylbenzaldehyde (HL) | [Co_2_L_2_Cl_2_(CH_3_OH)_2_] | -4.22^b^ | 99.461 | [[31](#_ENREF_31)] |
| 2,2’-bipyrimidine | [(Co_2_(*μ*-OH_2_)(*μ*-C(CH_3_)_3_COO)_2_  (C(CH_3_)_3_COO)_2_][(*μ*-bipym)]_n_ | -3^a^ | 108.7 | [[32](#_ENREF_32)] |
| 2,6-bis-[N,N-di(2- pyridylmethyl)aminomethyl]-  4-methylphenol (HL2) diphenylphosphinate (DPP) | [Co_2_(L2)(*μ*_2_-BNPP)_2_](ClO_4_).2CH_3_CN | -3.09^a^ | 126.05 | [[33](#_ENREF_33)] |
| *N*,*N*,*N*′,*N*′-tetramethylethylenediamine  benzohydroxamate anion | [Co_3_(*μ*-CF_3_COO)_4_(μ-BA)_2_(tmen)_2_] | -3.1^a^ | 118.5 | [[18](#_ENREF_18), [34](#_ENREF_34)] |
| 2,6-bis((4-(pyridin-2-yl)pyrimidin-2-ylthio)methyl)-4-methylphenol H(bpmp) | [Co_2_(bpmp)(*μ*-CH_3_COO)(CH_3_COO)_2_] .3H_2_O | -3.63^a^ | 120.0 | [[35](#_ENREF_35)] |
| (2E,7E,10E,15E)-1^5^,9^5^-dimethyl-3,7,11,15-tetraaza-1,9(1,3)-dibenzenacyclohexadecaphane-2,7,10,15-tetraene-1^2^,9^2^-diol | [Co_2_(tidf)(ClO_4_)_2_(H_2_O)_2_] | -10.3^b^ | 98.15 | [[36](#_ENREF_36)] |
| *N*,*N*,*N*′,*N*′-tetramethylethylenediamine  acetohydroxamate anion | [Co_3_(*μ*-CF_3_COO)_4_(μ-AA)_2_(tmen)_2_] | -6.4^a^ | 119.31 | [[18](#_ENREF_18), [34](#_ENREF_34), [37](#_ENREF_37)] |
| *N*, *N*, *N*’, *N*’- tetrakis(2 -benzimidazolylmethyl)-2-hydroxyl -1,3-diaminopropane  (HL1)  diphenylphosphinate (DPP) | [Co_2_(L1)(*μ*_2_-DPP)](ClO_4_)_2_. 0.5CH_3_CN.0.5C_2_H_5_OC_2_H_5_.H_2_O | -13.0^a^ | 127.4 | [[33](#_ENREF_33)] |
| 1,3-bis(bis(pyridin-2-ylmethyl)amino)propan-2-ol (HL^1^) | [Co_2_L^1^(OAc)](ClO_4_)_2_·0.5 H_2_O | -14.9^a^ | 123.26 | [[38](#_ENREF_38)]  This work |

^a^ *H* = -*2J_ex_S_1_.S_2_* ; ^b^ *H* = -*J_ex_S_1_.S_2_*

Figure S1. Relationship between J (cm^-1^) and Ni-O-Ni angle (°);[Ni_2_(L1)(OAc)(H_2_O)_2_](ClO_4_)_2_·H_2_O data shown as ⃰

Figure S1. Relationship between J (cm^-1^) and Ni-O-Ni angle (°);[Co_2_(L1)(OAc)](ClO_4_)_2_·H_2_O data shown as ⃰

**Computations**

The Cartesian coordinates for the optimized geometries for the four complexes are listed below, together with the following energies (in Hartree):

B3LYP solution-phase electronic energy (E), and

B3LYP solution-phase Gibbs free energy at 298.15 K and 1 mol L^–1^ (G)

[Ni_2_(L1)(μ-BDNPP)(H_2_O)]^2+^

-3864.5729558

-3863.896914

C -0.629303 -3.696999 0.187905

H 0.185022 -3.068258 0.527964

C -0.812495 -4.977085 0.705668

H -0.131609 -5.351836 1.462037

C -1.883728 -5.736448 0.239083

H -2.064201 -6.735937 0.623502

C -2.731820 -5.190868 -0.725990

H -3.580519 -5.752612 -1.104296

C -2.475567 -3.906997 -1.203887

C -3.314157 -3.271812 -2.298716

H -2.834652 -3.479365 -3.261137

H -4.316513 -3.722656 -2.320674

C -3.743415 -1.092542 -3.381466

H -4.824541 -0.903614 -3.425365

H -3.508907 -1.749882 -4.226918

C -2.978959 0.205072 -3.589461

C -3.435316 1.181640 -4.475081

H -4.399280 1.066680 -4.961977

C -2.629583 2.290095 -4.735154

H -2.962467 3.055990 -5.429465

C -1.395647 2.398954 -4.094208

H -0.728794 3.237044 -4.262602

C -1.023243 1.398503 -3.201947

H -0.085400 1.443369 -2.661618

N -1.437862 -3.177674 -0.746829

N -3.377434 -1.799866 -2.134665

N -1.796066 0.326834 -2.959282

O -2.314277 -0.340580 0.010942

O -0.386895 -2.221495 -3.276886

Ni -1.405955 -1.200495 -1.545651

C -1.504873 3.369369 -0.385781

H -0.503500 2.994239 -0.565653

C -1.949827 4.541463 -0.990757

H -1.289562 5.095487 -1.649724

C -3.249978 4.971006 -0.725969

H -3.639467 5.876961 -1.181558

C -4.041346 4.224042 0.143721

H -5.053727 4.535990 0.383790

C -3.506924 3.074756 0.731873

C -4.265852 2.334669 1.819518

H -4.053739 2.871309 2.748563

H -5.349509 2.391676 1.635439

C -4.447839 -0.036039 1.097558

C -3.900918 0.520713 3.434525

H -4.932472 0.304817 3.750170

H -3.495949 1.358207 4.011970

C -2.990054 -0.655899 3.720776

C -3.284842 -1.606539 4.698817

H -4.237300 -1.572399 5.219440

C -2.334144 -2.580685 5.006198

H -2.539065 -3.322841 5.772509

C -1.119877 -2.581590 4.321185

H -0.343933 -3.310205 4.531521

C -0.911677 -1.611845 3.342219

H 0.015545 -1.563637 2.781746

N -2.265112 2.650500 0.448539

N -3.826051 0.940693 2.015443

N -1.824181 -0.678267 3.048168

O -1.520227 2.219152 3.120054

Ni -1.709546 0.950806 1.656919

C -4.225932 -1.417233 -0.966630

C -3.675008 -0.177019 -0.234044

H -4.196237 -2.256461 -0.265936

H -5.269010 -1.282189 -1.288965

H -5.505259 0.215365 0.916011

H -4.418677 -1.008308 1.599253

H -3.872300 0.718807 -0.854220

O 3.825795 -3.784474 2.878045

O 1.858538 -3.152681 2.184602

O 0.368016 0.614893 1.196743

O 5.194896 5.379286 2.952709

N 3.080332 -3.276238 2.051230

H 4.141338 3.221419 2.951762

C 3.799096 3.279986 1.925672

H 2.907571 1.329881 1.911979

N 4.822863 5.531335 1.792545

C 3.105541 2.227035 1.339874

C 4.072194 4.422625 1.177040

O 5.023254 6.526361 1.101176

O 2.336359 -0.868518 0.756712

C 2.657048 2.321568 0.016790

P 1.161903 0.075911 0.058142

C 3.655212 4.545936 -0.139502

O 2.052803 1.286419 -0.645118

C 3.681234 -2.830626 0.785690

C 2.927354 3.503004 -0.702529

H 3.878906 5.437317 -0.711373

O 3.158270 4.328132 -2.842386

C 3.239227 -1.666866 0.124349

N 2.438150 3.707152 -2.070879

H 5.049296 -4.496470 0.797035

C 4.698343 -3.622051 0.263768

O 0.582728 -0.683261 -1.114251

O 1.313440 3.278667 -2.352100

C 3.827684 -1.333249 -1.103654

C 5.242123 -3.272251 -0.963761

H 3.519618 -0.427241 -1.610959

C 4.817362 -2.139173 -1.654995

O 6.635701 -5.113422 -0.905516

N 6.294243 -4.123496 -1.546377

H 5.278492 -1.892373 -2.603830

O 6.749537 -3.783395 -2.635371

H -0.726726 2.005612 3.632237

H 0.402416 -2.558313 -2.819246

H -0.055640 -1.480145 -3.811349

[Ni_2_(L1)(BDNPP)(H_2_O)_2_(OH)]^+^

-3941.0196194

-3940.317070

C 2.431654 -1.640098 -2.917822

H 1.876027 -0.710708 -2.998551

C 3.221911 -2.131352 -3.955866

H 3.252807 -1.609011 -4.906227

C 3.966041 -3.290246 -3.739740

H 4.595621 -3.697669 -4.525430

C 3.907533 -3.915850 -2.493046

H 4.492523 -4.807393 -2.287748

C 3.078157 -3.379826 -1.509296

C 2.907544 -4.034109 -0.146193

H 2.043188 -4.702939 -0.193684

H 3.789478 -4.644496 0.094216

C 1.902458 -3.574741 2.070269

H 2.592803 -3.988382 2.818927

H 1.280724 -4.403114 1.714250

C 0.974569 -2.557091 2.713128

C 0.568693 -2.670959 4.041987

H 0.983209 -3.451996 4.672457

C -0.391049 -1.786274 4.534310

H -0.735429 -1.869749 5.560868

C -0.907731 -0.805592 3.689196

H -1.676770 -0.112485 4.011204

C -0.429591 -0.739424 2.384291

H -0.795294 0.000751 1.684579

N 2.350458 -2.269084 -1.736115

N 2.633261 -3.026238 0.907522

N 0.488235 -1.593482 1.909096

O 2.828383 -0.317458 0.377352

O -0.077305 -3.255488 -0.472050

Ni 1.313617 -1.605651 -0.033979

C 0.760676 2.585535 1.440061

H 0.264019 2.471024 0.481554

C 0.070505 2.974319 2.584254

H -0.990357 3.197393 2.540208

C 0.774451 3.052966 3.787340

H 0.269530 3.344593 4.703587

C 2.133571 2.749805 3.797945

H 2.708815 2.800150 4.718113

C 2.759849 2.394806 2.600011

C 4.267881 2.182535 2.552577

H 4.734670 3.174444 2.565651

H 4.597056 1.678143 3.472362

C 4.903471 0.001651 1.554768

C 5.974829 2.050912 0.772915

H 6.870878 1.779469 1.351476

H 5.875060 3.141381 0.813304

C 6.144552 1.661046 -0.685902

C 7.389310 1.645716 -1.314156

H 8.293725 1.839997 -0.745070

C 7.443410 1.394725 -2.685751

H 8.399330 1.385172 -3.201353

C 6.257935 1.166409 -3.384597

H 6.261842 0.986184 -4.454555

C 5.055882 1.180058 -2.680188

H 4.084171 1.035015 -3.142364

N 2.076121 2.313052 1.447274

N 4.743860 1.465948 1.353021

N 5.013007 1.408704 -1.361658

Ni 3.216687 1.599303 -0.232679

C 3.858986 -2.264770 1.285838

C 3.563743 -0.757538 1.468425

H 4.563863 -2.359875 0.454563

H 4.322632 -2.713913 2.176620

H 5.417778 -0.199930 2.508131

H 5.547273 -0.354807 0.745693

H 3.015468 -0.611400 2.419532

O -4.020536 -2.731563 2.501051

O -2.256256 -3.120950 1.299009

O -1.781966 0.047365 -2.895220

O -6.441001 5.214622 -2.297596

N -3.430460 -2.743822 1.430378

H -4.664139 3.831354 -3.123191

C -4.400900 3.416239 -2.157736

H -2.771661 2.196719 -2.885964

N -6.202128 4.798897 -1.167033

C -3.354191 2.509568 -2.027963

C -5.110142 3.819989 -1.026254

O -6.795608 5.130703 -0.143768

O -2.236419 -1.356050 -0.729302

C -3.038892 1.980833 -0.769733

P -1.410538 -0.090141 -1.466418

C -4.809145 3.333481 0.238544

O -1.953243 1.189333 -0.542701

C -4.176532 -2.333633 0.239351

C -3.791516 2.393816 0.346650

H -5.360236 3.667533 1.108538

O -3.483122 2.634633 2.620456

C -3.558529 -1.654820 -0.834181

N -3.502197 1.846396 1.675159

H -5.999702 -3.139869 1.060287

C -5.532630 -2.643625 0.219474

O 0.003063 -0.273348 -0.972943

O -3.307999 0.634401 1.768865

C -4.336566 -1.332202 -1.956256

C -6.269315 -2.309304 -0.906210

H -3.860899 -0.829148 -2.789667

C -5.685931 -1.662485 -1.995012

O -8.166927 -3.240967 0.025577

N -7.700720 -2.652756 -0.946886

H -6.293748 -1.427028 -2.860460

O -8.327160 -2.325339 -1.951415

O 3.310487 3.734167 -1.004813

H 2.676032 3.216662 -1.606742

H -0.924970 -3.087736 -0.002399

H -0.296115 -3.173109 -1.413958

H 2.738597 4.329623 -0.495043

O 2.135655 1.679054 -1.865633

H 1.255647 1.307958 -1.703858

**References**

[1] K.K. Nanda, L.K. Thompson, J.N. Bridson, K. Nag, J. Chem. Soc., Chem. Commun., (1994) 1337.

[2] K.K. Nanda, R. Das, L.K. Thompson, K. Venkatsubramanian, P. Paul, K. Nag, Inorg. Chem., 33 (1994) 1188.

[3] A. Biswas, M.G.B. Drew, C.J. Gomez-Garcia, A. Ghosh, Polyhedron, 121 (2017) 80.

[4] K. Mochizuki, A. Hasegawa, T. Weyhermuller, Inorg. Chim. Acta, 357 (2004) 3245.

[5] A. Biswas, L.K. Das, M.G.B. Drew, G. Aromi, P. Gamez, A. Ghosh, Inorg. Chem., 51 (2012) 7993.

[6] K.K. Nanda, R. Das, L.K. Thompson, K. Venkatsubramanian, K. Nag, Inorg. Chem., 33 (1994) 5934.

[7] Y.-w. Ren, J.-x. Lu, B.-w. Cai, D.-b. Shi, H.-f. Jiang, J. Chen, D. Zheng, B. Liu, Dalton Trans., 40 (2011) 1372.

[8] S.S. Massoud, C.C. Ledet, T. Junk, S. Bosch, P. Comba, R. Herchel, J. Hosek, Z. Travnicek, R.C. Fischer, F.A. Mautner, Dalton Trans., 45 (2016) 12933.

[9] P.L. Pawlak, A.Y.S. Malkhasian, B. Sjlivic, M.J. Tiza, B.E. Kucera, R. Loloee, F.A. Chavez, Inorg. Chem. Commun., 11 (2008) 1023.

[10] A. Greatti, M. Scarpellini, R.A. Peralta, A. Casellato, A.J. Bortoluzzi, F.R. Xavier, R. Jovito, M. Aires de Brito, B. Szpoganicz, Z. Tomkowicz, M. Rams, W. Haase, A. Neves, Inorg. Chem., 47 (2008) 1107.

[11] H. Adams, D. Bradshaw, D.E. Fenton, Inorg. Chim. Acta, 332 (2002) 195.

[12] T. Chattopadhyay, M. Mukherjee, A. Mondal, P. Maiti, A. Banerjee, K.S. Banu, S. Bhattacharya, B. Roy, D.J. Chattopadhyay, T.K. Mondal, M. Nethaji, E. Zangrando, D. Das, Inorg. Chem., 49 (2010) 3121.

[13] S. Mandal, V. Balamurugan, F. Lloret, R. Mukherjee, Inorg. Chem., 48 (2009) 7544.

[14] H.E. Wages, K.L. Taft, S.J. Lippard, Inorg. Chem., 32 (1993) 4985.

[15] R.M. Buchanan, M.S. Mashuta, K.J. Oberhausen, J.F. Richardson, Q. Li, D.N. Hendrickson, J. Am. Chem. Soc., 111 (1989) 4497.

[16] P. Chaudhuri, H.J. Kueppers, K. Wieghardt, S. Gehring, W. Haase, B. Nuber, J. Weiss, J. Chem. Soc., Dalton Trans., (1988) 1367.

[17] B.E. Schultz, B.-H. Ye, X.-y. Li, S.I. Chan, Inorg. Chem., 36 (1997) 2617.

[18] Z. Tomkowicz, S. Ostrovsky, S. Foro, V. Calvo-Perez, W. Haase, Inorg. Chem., 51 (2012) 6046.

[19] L.J. Daumann, J.A. Larrabee, P. Comba, G. Schenk, L.R. Gahan, Eur. J. Inorg. Chem., (2013) 3082.

[20] S. Ostrovsky, K. Falk, J. Pelikan, D.A. Brown, Z. Tomkowicz, W. Haase, Inorg. Chem., 45 (2006) 688.

[21] D.A. Brown, W. Errington, W.K. Glass, W. Haase, T.J. Kemp, H. Nimir, S. Ostrovsky, R. Werner, Inorg. Chem., 40 (2001) 5962.

[22] A. Jana, S. Konar, K. Das, S. Ray, J.A. Golen, A.L. Rheingold, L.M. Carrella, E. Rentschler, T.K. Mondal, S.K. Kar, Polyhedron, 38 (2012) 258.

[23] O. Fabelo, L. Canadillas-Delgado, J. Pasan, F.S. Delgado, F. Lloret, J. Cano, M. Julve, C. Ruiz-Perez, Inorg. Chem., 48 (2009) 11342.

[24] H. Arora, S.K. Barman, F. Lloret, R. Mukherjee, Inorg. Chem., 51 (2012) 5539.

[25] M. Hossain, M. Yamasaki, M. Mikuriya, A. Kuribayashi, H. Sakiyama, Inorg. Chem., 41 (2002) 4058.

[26] H. Sakiyama, J. Comput. Chem. Jpn., 6 (2007) 123.

[27] V. Calvo-Perez, S. Ostrovsky, A. Vega, J. Pelikan, E. Spodine, W. Haase, Inorg. Chem., 45 (2006) 644.

[28] D.A. Brown, W.K. Glass, N.J. Fitzpatrick, T.J. Kemp, W. Errington, G.J. Clarkson, W. Haase, F. Karsten, A.H. Mahdy, Inorg. Chim. Acta, 357 (2004) 1411.

[29] F.P. Pruchnik, U. Dawid, A. Kockel, Polyhedron, 25 (2006) 3647.

[30] S. Ostrovsky, Z. Tomkowicz, W. Haase, Inorg. Chem., 49 (2010) 6942.

[31] S.-Y. Lin, G.-F. Xu, L. Zhao, J. Tang, G.-X. Liu, Z. Anorg. Allg. Chem., 637 (2011) 720.

[32] P. Albores, E. Rentschler, Dalton Trans., (2009) 2609.

[33] J.-L. Tian, W. Gu, S.-P. Yan, D.-Z. KLiao, Z.-H. Jiang, Zeit. Anorg. Allge. Chemie. , 634 (2008) 1775.

[34] Z. Tomkowicz, S. Ostrovsky, H. Muller-Bunz, A.J. Hussein Eltmimi, M. Rams, D.A. Brown, W. Haase, Inorg. Chem., 47 (2008) 6956.

[35] C. Huang, G. Xu, H. Zhu, Y. Song, S. Gou, Inorg. Chim. Acta, 361 (2008) 5.

[36] R.B. Samulewski, J.C. da Rocha, O. Fuganti, R. Stieler, E.S. Lang, M.G.F. Vaz, F.S. Nunes, J. Mol. Struct., 984 (2010) 354.

[37] D.A. Brown, G.J. Clarkson, N.J. Fitzpatrick, W.K. Glass, A.J. Hussein, T.J. Kemp, H. Muller-Bunz, Inorg. Chem. Commun., 7 (2004) 495.

[38] G.S. Siluvai, N.N. Murthy, Inorg. Chim. Acta, 362 (2009) 3119.
